# A field guide to flow chemistry for synthetic organic chemists†

**DOI:** 10.1039/d3sc00992k

**Published:** 2023-03-15

**Authors:** Luca Capaldo, Zhenghui Wen, Timothy Noël

**Affiliations:** a Flow Chemistry Group, Van ‘t Hoff Institute for Molecular Sciences (HIMS), University of Amsterdam 1098 XH Amsterdam The Netherlands t.noel@uva.nl

## Abstract

Flow chemistry has unlocked a world of possibilities for the synthetic community, but the idea that it is a mysterious “black box” needs to go. In this review, we show that several of the benefits of microreactor technology can be exploited to push the boundaries in organic synthesis and to unleash unique reactivity and selectivity. By “lifting the veil” on some of the governing principles behind the observed trends, we hope that this review will serve as a useful field guide for those interested in diving into flow chemistry.

## Abstract 2

This review explores the benefits of flow chemistry and dispels the notion that it is a mysterious “black box”, demonstrating how it can push the boundaries of organic synthesis through the understanding of its governing principles.

## Graphical abstract

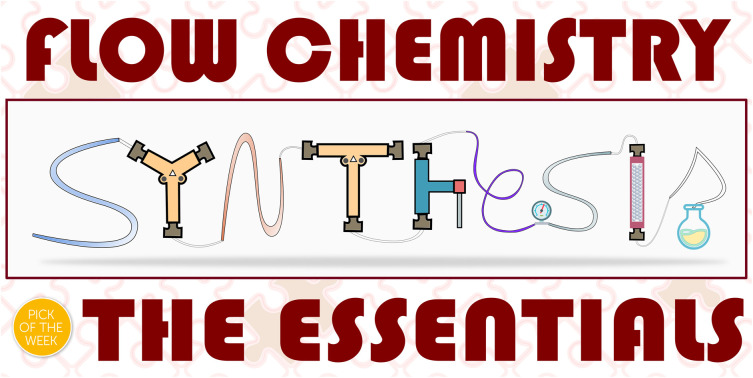

## 1. Introduction

Flow chemistry is a discipline in synthetic organic chemistry that uses a continuous stream of different reagents, which are introduced by pumps and mixed in a continuous reactor, such as a plug flow reactor (PFR) or continuous-stirred tank reactor (CSTR).^1,2^ Compared to conventional batch processing which is often carried out in round-bottom flasks, it offers several advantages such as enhanced mass and heat transfer, improved safety, increased reaction efficiency, reduced waste, better scalability, and improved reproducibility.^3,4^ As a consequence, flow chemistry allows for precise control over reaction conditions and enables real-time monitoring and analysis of reaction kinetics, resulting in high-quality products and streamlined processes. These benefits have led to the increasing adoption of flow chemistry in academia and various industries for pharmaceuticals, fine chemicals, and materials science.^5–8^

While undoubtedly flow chemistry has numerous advantages, it was received with the skepticism of the synthetic community,^9^ therefore its implementation experienced an induction period. This can be attributed to a lack of interdisciplinary knowledge, perceived complexity, and high investment costs (see ESI†). Indeed, flow chemistry is an interdisciplinary field that requires knowledge from both chemistry and chemical engineering. However, some basic understanding of these principles of flow chemistry should already allow one to begin setting up flow experiments. Furthermore, recent advancements in “Do It Yourself”-assembled flow setups,^10–12^ 3D-printing technology,^13^ and cheap electronic toolkits^14^ have made technology more intuitive, accessible and affordable. As a consequence, adoption of flow technology in synthetic organic chemistry has been growing in recent years. With the rise of photo- and electrochemistry, flow technology has become a popular and indispensable choice due to its capability to handle the scalability challenges of these synthetic modes. Flow chemistry is also favored for its ability in safely and effectively conducting reactions with challenging or hazardous reagents, expanding the chemical frontiers.

Frequently, our lab gets asked to help young MSc and PhD students start using the technology.^15,16^ Although they may feel intimidated at first, we often see how quickly they grasp the concepts and start reaping the benefits of flow technology for their research. To further increase the adoption of flow chemistry in synthetic organic chemistry, this review seeks to provide some basic guidelines for the use of continuous-flow reactors. The goal is to deliver a concise overview to help researchers gain a basic understanding of the principles behind this technology, allowing them to get the most out of their experiments. We have highlighted three relevant examples to clarify each fundamental principle. Our aim is not to provide an exhaustive overview of continuous-flow chemistry,^17^ but rather to offer simple and easy-to-follow guidelines for readers to determine if flow chemistry is relevant to their research. Consequently, our objective is to educate the broader synthetic community about this innovative technology and demonstrate how and when it can make a difference.

## 2. Mass transfer

The first, and arguably most potent, advantage of flow chemistry for synthetic organic chemists is the improved mass transfer. Mass transfer is defined as the net movement of one species, *e.g.* one of the reactants, from one point to another within the reactor due to diffusion and/or convection. In other words, mass transfer defines the degree of mixing in the reaction mixture: the better the mass transfer, the more efficient the mixing.

This parameter is especially crucial in the case of multiphase reactions, *e.g.* gas–liquid reactions where one of the reagents needs to migrate by diffusion from one phase to another.^18^

**Fig. 1:**
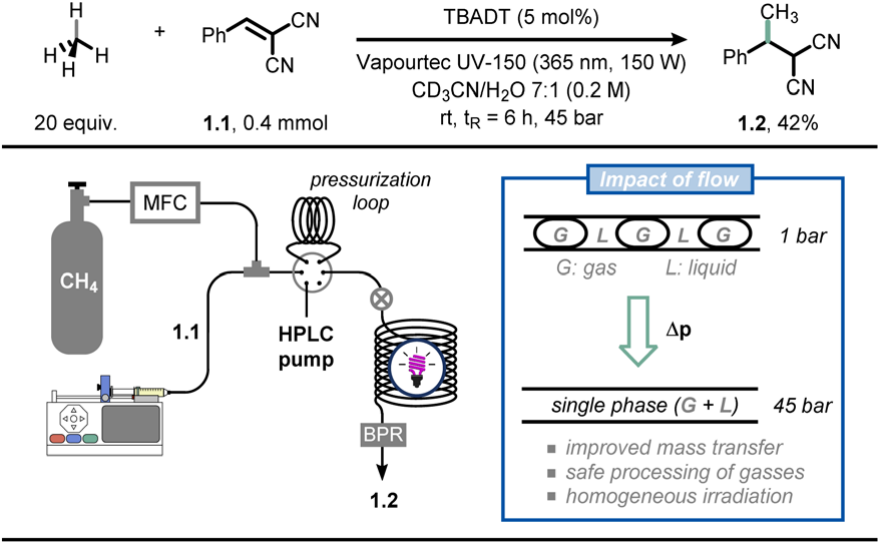
Microflow technology improves gas–liquid mass transfer, which can be exploited for the functionalization of gaseous light hydrocarbons. TBADT: tetrabutylammonium decatungstate; BPR: back-pressure regulator; MFC: mass-flow controller.

As an example, Noël and co-workers reported the photocatalytic Giese-type alkylation using gaseous light hydrocarbons (*i.e.*, methane, ethane, propane, isobutane) *via* hydrogen atom transfer photocatalysis in flow (Fig. 1).^19^ Hereto, the authors exploited the decatungstate anion (DT, W_10_O_32_^4−^) as a versatile and inexpensive polyoxometalate-based hydrogen atom transfer (HAT) photocatalyst:^20^ upon activation by UV-light irradiation, this photocatalyst can cleave homolytically C(sp^3^)–H bonds to yield C-centered radicals which can be subsequently exploited for various synthetic purposes.^21^ While this chemistry is documented to be efficient in the case of homogeneous solutions (*i.e.*, single solution phase), the activation of gaseous alkanes is more challenging due to their limited solubility in common organic solvents. The immediate consequence is that the targeted chemistry is particularly slow due to poor gas-to-liquid mass transfer limitations. The authors tackled this challenge by resorting to flow chemistry: by increasing the pressure in the reactor through use of simple back-pressure regulators, the gaseous alkanes could be forced into the liquid phase, increasing the odds of C(sp^3^)–H bond activation of the gaseous components. Thus, when a CD_3_CN : H_2_O (7 : 1) solution of olefin 1.1 was irradiated with UV light (365 nm, 150 W) in the presence of tetrabutylammonium decatungstate and methane (20 equiv.) at a pressure of 45 bar, the corresponding methylated product 1.2 was obtained in 42% yield after 6 hours residence time. Intriguingly, flow chemistry allowed to conduct the entire scope (38 examples) at high pressure in a timely and scalable yet safe fashion, which is by no means possible in conventional batch reactors. Very recently, the same authors extended this technology for the C(sp^3^)–H carbonylation with gaseous carbon monoxide (CO), obtaining unsymmetrical ketones (41 examples) in good to excellent yields.^22^

**Fig. 2:**
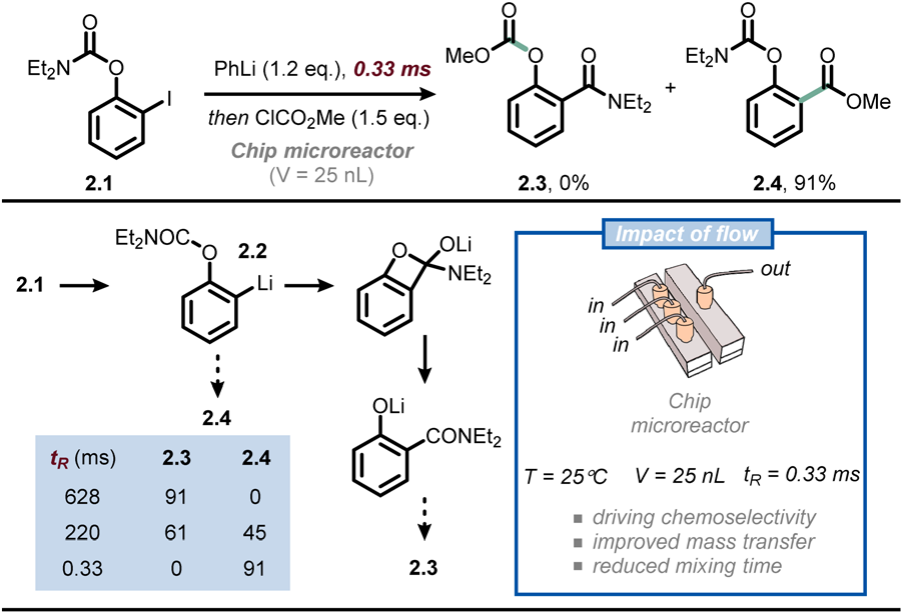
Ultra-fast mixing provided by flow technology allows to outpace undesired anionic Fries rearrangement in the functionalization of iodophenyl carbamates at the *ortho* position. *t*_R_: residence time.

Another synthetic discipline that relies heavily on the optimal mass transfer offered by microflow technology is that of flash chemistry.^23,24^ Flash chemistry can be considered a subdiscipline of flow chemistry, where extremely fast reactions are conducted in a highly controlled manner to produce the desired compounds with high selectivity. In 2016, Yoshida, Kim and co-workers exploited this concept to outpace the very rapid anionic Fries rearrangement for the chemoselective functionalization of iodophenyl carbamates at the *ortho* position (Fig. 2).^25^ Thus, when compound 2.1 is subjected to iodine/lithium exchange, intermediate 2.2 is obtained; the latter compound rapidly undergoes anionic Fries rearrangement at room temperature to give 2.3. To outpace this rearrangement and functionalize the *ortho* position of 2.2 with an electrophile, the authors developed a chip microreactor with a 3D serpentine microchannel design made of six layers of UV-laser-ablated fluoroethylene propylene-polyimide films. The chemically inert reactor is capable of withstanding high pressure and low reaction temperatures, while its volume is merely 25 nanoliters. Such reduced internal volume enables exceedingly fast mixing times (as low as 330 ms), which is crucial to quench 2.2 with a suitable electrophile to yield 2.4 before its premature rearrangement. When prolonging the mixing time to the millisecond range, the selectivity was already reversed in favor of product 2.3. The same technology was successfully used for the synthesis of Afesal, a biologically active compound having anthelmintic activity, with a productivity of 5.3 g h^−1^. Overall, flow chemistry allowed to steer reactivity favoring an intermolecular reaction over an intramolecular one, which is not possible in batch.

**Fig. 3:**
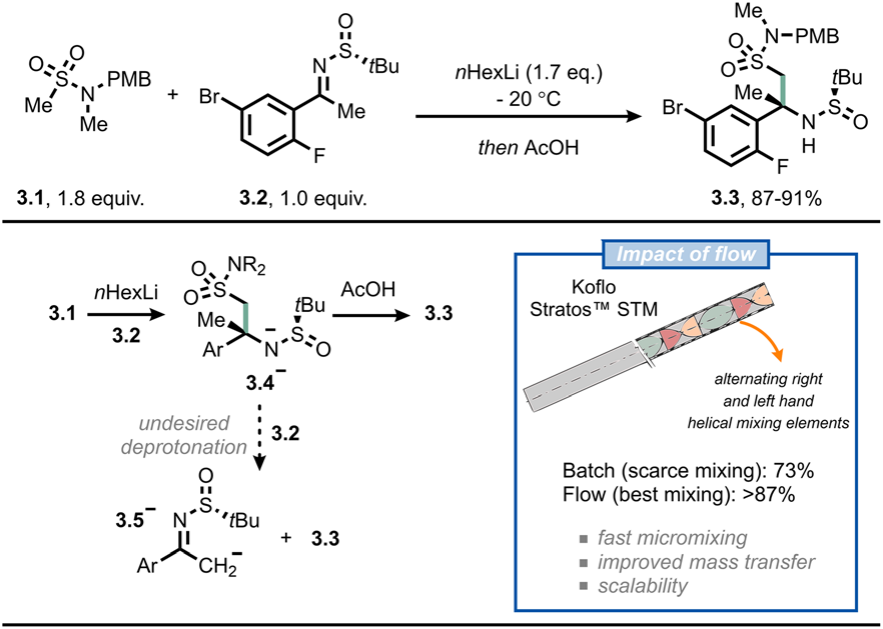
Adoption of flow chemistry in an industrial setting allows to avoid undesired fast deprotonation in the synthesis of 3.3, an intermediate for the manufacturing of verubecestat (MK-8931). STM: static tube mixer.

A third example where flow chemistry was adopted to tackle an issue related to mass transfer was reported by scientists from Merck for the synthesis of verubecestat (MK-8931).^26^ This drug was projected to be a breakthrough drug for Alzheimer's disease. However, during late-stage trials, the compound turned out to be not beneficial and displayed increased adverse effects.^27^ In their original synthetic approach, the crucial intermediate 3.3 was prepared in batch by reacting the organolithium derived from compound 3.1 with sulfinamide 3.2 under cryogenic conditions (≤60 °C) with moderate assay yield (73%). According to the authors, the reason for this modest result was the inefficient mixing: in fact, once anion 3.4^−^ is formed, it tends to overreact by deprotonating 3.2 to give 3.5^−^ (Fig. 3). This would eventually lead to disguised selectivity and diminished yields; which is typical for reactions where the mixing time is longer than the reaction time.^28,29^ By switching to flow, and by incorporating different static mixing elements in their setup (Koflo Stratos™ mixers), the authors managed to steer the selectivity towards the desired product (3.3, 5 g h^−1^ based on assay yield) and outpace the fast deprotonation of the electrophile. A few years later, the researchers were able to scale the process up to pilot-plant scale by relying on flow chemistry.^30^

## 3. Heat transfer

The high area-to-volume ratio of microchannels makes heat transfer much more efficient than in conventional reactors, such as round-bottom flasks. With a large heat exchange surface, hot spots can be prevented and the dangers of thermal runaways mitigated.^31–33^ The main benefits of efficient heat transfer in flow chemistry for organic synthesis are the ability to operate under isothermal conditions and to operate under superheated conditions.

**Fig. 4:**
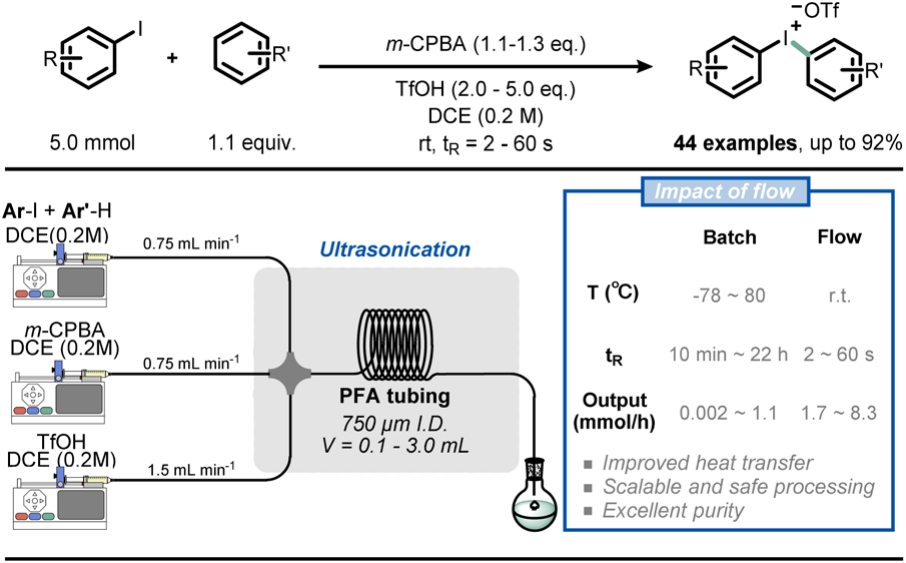
Use of flow technology for the handling of the exothermic synthesis of diaryliodonium triflates. *m*-CPBA: *meta*-chloroperoxybenzoic acid.

The nearly isothermal behaviour of microreactors allows chemists to precisely control the temperature of the reaction, resulting in improved chemical selectivity and safer handling of exothermic reactions, such as nitration,^34,35^ halogenation,^36,37^ and organometallic-based reactions.^38,39^ For example, Noël and co-workers reported a safe and scalable synthesis of diaryliodonium triflates under flow conditions (Fig. 4).^40^ Diaryliodonium salts have been widely used as aryl electrophile sources in many arylation reactions;^41^ however, the reaction is highly exothermic, which poses a significant safety risk when carried out on a large scale. By adopting flow conditions, such exothermic reactions can be performed safely due to the efficient heat exchange. In this way, with residence times varying from 2 to 60 seconds and room temperature conditions, the authors obtained different diaryliodonium salts (44 examples) on a gram scale from a wide range of electron-rich and electron-deficient arenes.

**Fig. 5:**
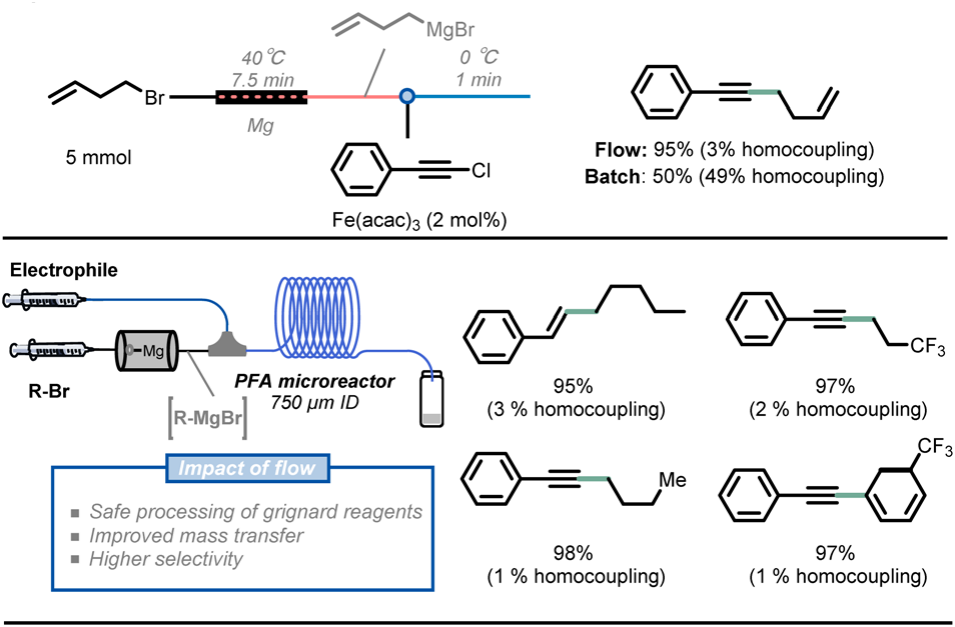
Combining Grignard reagent synthesis with iron-catalyzed cross-coupling reaction in a telescoped flow process for C(sp)–C(sp^3^) bond formation.

Similarly, Alcazar *et al.* reported the direct preparation of Grignard reagents at 40 °C using a magnesium-packed-bed reactor in flow, which greatly expanded the application of this powerful methodology.^42^ The synthesis of Grignard reagents typically requires low temperatures (<0 °C) to avoid the dangers of thermal runaways, but this type of synthesis can be easily achieved at room temperature in flow conditions. In addition, with the use of a magnesium-packed-bed flow reactor, freshly made solutions of Grignard reagents can be generated and immediately consumed in a subsequent transformation. As an example of this feature, Noël and co-workers developed a telescoped Fe-catalyzed C(sp)–C(sp^3^) cross-coupling transformation, resulting in higher yield and selectivity in flow (Fig. 5).^43^

**Fig. 6:**
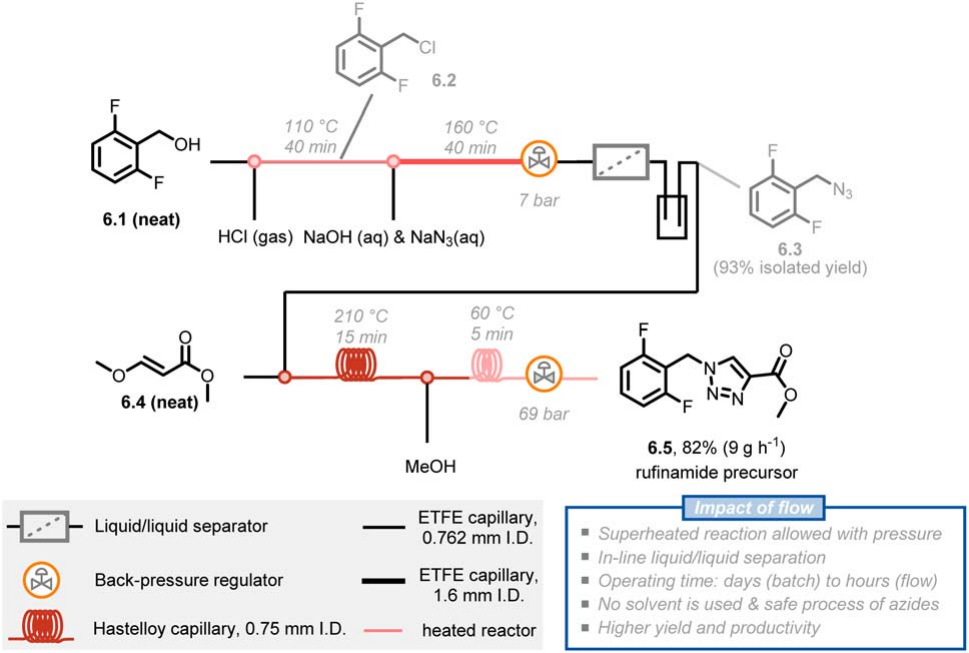
Schematic representation of a 5-stage multi-step flow synthesis of a rufinamide precursor under superheated conditions. ETFE: ethylene tetrafluoroethylene.

Another benefit of the enhanced heat transfer in flow reactors is the ability to handle superheated reactions at increased pressure, *i.e.* the increase of the reaction temperature above the atmospheric boiling temperature of the solvent. Many reactions are still slow even under reflux conditions, but the reaction rate can be further boosted under such superheated conditions using a simple back pressure regulator (BPR). For example, rufinamide, an important antiseizure medication, has been extensively reported in patents and literature and its synthesis benefits from this superheating effect.^33^ The formation of a 1,2,3-triazole precursor 6.5 through a 1,3-dipolar Huisgen cycloaddition of 2,6-difluorobenzylazide 6.3 with an appropriate dipolarophile is a key step in the synthesis of rufinamide. Mudd and Stevens utilized a nontoxic and inexpensive (*E*)-methyl 3-methoxyacrylate 6.4 as a dipolarophile, obtaining the desired 1,4-cycloadduct.^44^ However, this method requires 28 hours of reaction time to achieve full conversion at 135 °C and under solvent-free conditions, which poses a safety concern about runaway decomposition with gas formation and concomitant pressure build-up during batch processing. Noël and Hessel presented an intensified method with 210 °C and 69 bar reaction conditions, significantly boosting the reaction rate and obtaining the targeted 1,2,3-triazole precursor 6.5 in only 10 minutes of residence time.^45^ Furthermore, a 5-stage 3-step continuous synthesis with integrated separation steps delivered a total yield of 82% and productivity of 9 g h^−1^ of rufinamide precursor. The process minimizes the isolation and handling of energetic intermediates while minimizing water and organic solvent consumption (Fig. 6).^46^

## 4. Multi-step synthesis

The possibility to streamline several transformations in a “single, continuous and uninterrupted reactor network”^47^ is a riveting opportunity offered by flow chemistry. In fact, multi-step syntheses in batch are tedious, time-consuming and labor-intensive undertakings. For example, the product of one step must often be purified before the subsequent one can be executed. In contrast, organic chemists can use a one-flow, multi-step synthesis approach to expedite their synthetic routes. However, to be successful, some major challenges have to be tackled, such as solvent compatibilities and byproduct formation, requiring often inline purification strategies.

**Fig. 7:**
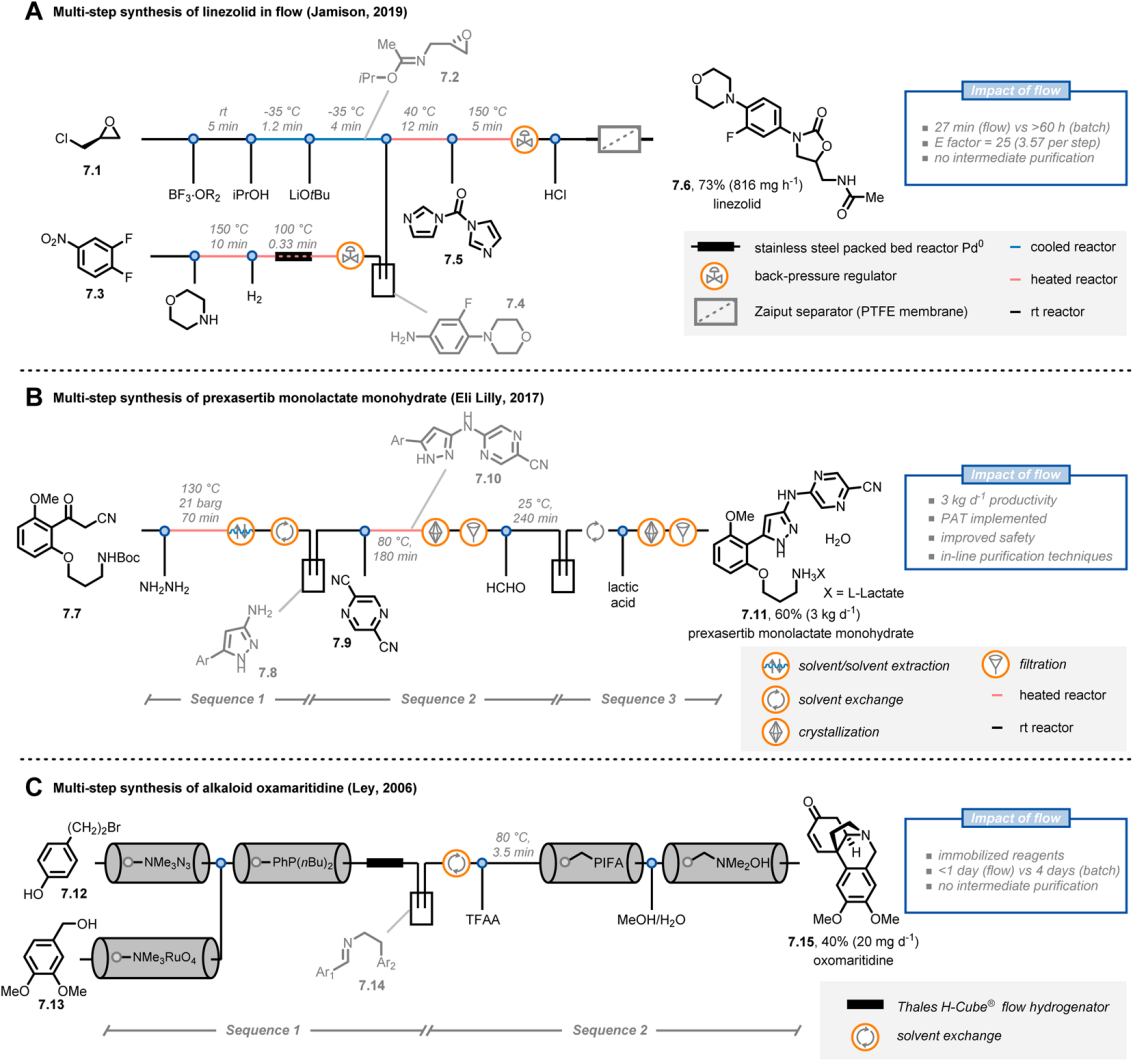
Application of flow chemistry for multi-step synthesis. PAT: process analytical technology. TFAA: trifluoroacetic anhydride.

Jamison and co-workers reported the continuous-flow synthesis of the antibiotic linezolid by combining seven consecutive steps without purification of any intermediate (Fig. 7A).^48^ Notably, all of the steps are compatible with each other, requiring neither solvent exchanges nor intermediate work-ups. The entire process involves a convergent synthesis based on three modules. In the first module, (+)-epichlorohydrine 7.1 was reacted with boron trifluoride etherate as a stoichiometric Lewis acid in the presence of acetonitrile to obtain the corresponding Ritter product; next, epoxide formation was achieved under basic conditions to get 7.2. Second, in parallel with the first module, aniline 7.4 was obtained from nitroarene 7.3*via* S_N_Ar with morpholine and subsequent heterogeneous hydrogenation with a palladium packed-bed reactor. In the third module, the streams containing 7.2 and 7.4 were combined to afford linezolid (7.6) after reaction with *N*,*N*-carbonyldiimidazole (7.5). Overall, linezolid was synthesized in 73% yield with a total residence time of 27 minutes, corresponding to a productivity of 816 mg h^−1^. This protocol showcases how a careful tuning of the reaction conditions and the flow parameters allows to accelerate the synthesis of pharmaceuticals: notably, the corresponding batch synthesis required more than 60 hours.

Interestingly, the concept of multi-step flow synthesis is also receiving a great deal of attention from the pharmaceutical industry.^49^ Continuous manufacturing has tremendous advantages over batch manufacturing in terms of improved performance and safety, together with decreased capital expenditures. This is even more true when the concept of small-volume continuous manufacturing (SVCM) is adopted. In such a scenario, small-size equipment that fits a flexible environment, such as a standard laboratory fumehood, is operated in a continuous fashion to reach productivities of several kg per day. For example, scientists at Eli Lilly reported the synthesis of prexasertib monolactate monohydrate, a checkpoint kinase 1 inhibitor, on a kilogram scale by taking advantage of the SVCM concept (Fig. 7B).^50^ Starting from compound 7.7, pyrazole 7.8 was prepared by reaction with hazardous NH_2_NH_2_: unlike batch, the flow approach required only a slight excess of hydrazine and allowed to minimize exposure to the operators. After a solvent exchange in an automated 20 L rotary evaporator, a S_N_Ar reaction was conducted with pyrazine 7.9 to afford compound 7.10, which was purified *via* a continuous crystallization using two mixed-suspensions, mixed-product removal vessels. In addition to being time-efficient, this custom purification solution eliminated the potential for operator exposure to 7.10 (for which the occupational exposure limit is 1 mg m^−3^). Finally, after Boc-deprotection and formation of the lactate salt, prexasertib monolactate monohydrate (7.11) was obtained. Overall, the researchers were able to produce 24 kg of the drug for use in human clinical trials. It is very important to stress that an extensive use of process analytical technologies (PATs) was implemented in the continuous-flow process allowing for informed process adjustments to ensure high end-product quality.

As a final example, Ley and colleagues reported the multi-step synthesis of the alkaloid oxomaritidine (7.15) by exploiting the concept of immobilized reagents, scavengers and catch/release techniques (Fig. 7C).^51,52^ Using this strategy,^53^ the authors managed to develop an automated flow sequence to yield oxomaritidine in less than a day. First, compound 7.12 was converted into the corresponding azide by exploiting an azide exchange resin; the exiting stream was directed over a second column containing a polymer-supported phosphine. The aza-Wittig intermediate was formed and retained on the resin in the column. In parallel, benzyl alcohol 7.13 was oxidized to the aldehyde by adopting a pre-packed column of tetramethylammonium perruthenate and the stream was subsequently passed through the column containing the aza-Wittig intermediate to form the targeted imine. The latter compound was then reduced and, after a manual solvent switch, the secondary amine was trifluoroacetylated. In a final sequence, oxidative phenolic coupling, *N*-deprotection and spontaneous cyclization afforded the targeted oxomaritidine. Interestingly, the use of immobilized reagents reduces the overall number of pumps needed for the platform, cutting the costs of the entire flow approach. However, one potential drawback of this strategy is that the productivity of the entire process is dependent on, and limited by, the amounts of immobilized reagents loaded in the packed-bed cartridges.

## 5. Photochemistry

Continuous-flow technology is often coupled with photochemistry and photocatalysis in synthetic campaigns, making it arguably the most popular application of flow chemistry.^54–59^ Light absorption is governed by the Beer–Lambert law, leading to a rapid decline of the light intensity when travelling through a reaction mixture that contains photon-absorbing molecules. Consequently, the center of the reactor receives almost no light, creating a “dark zone” where there is no reaction occurring. By exploiting microreactor technology, the entire reaction mixture experiences the same light intensity, leading to reduced reaction times, less deleterious side-product formation and high productivities.^56,58^

**Fig. 8:**
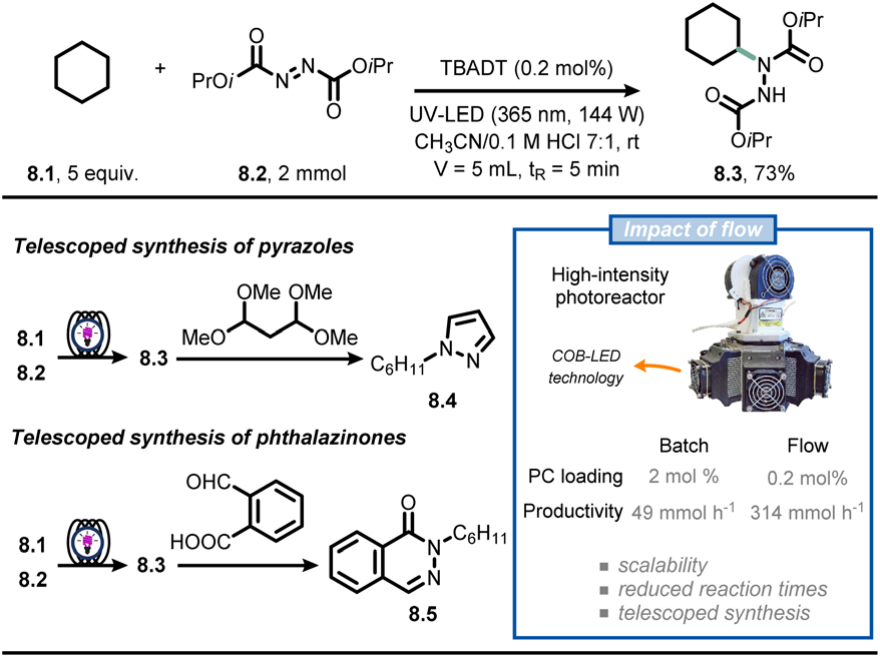
Flow technology-enabled high throughput for the formal photocatalytic amination of C(sp^3^)–H bonds.

Noël and co-workers reported on the use of flow chemistry to develop a scalable and accelerated protocol for the formal amination of C(sp^3^)–H bonds *via* decatungstate-photocatalyzed HAT (Fig. 8).^60^ To realize the C–N bond formation, the authors capitalized on the hydroalkylation of azadicarboxylates to deliver Boc-protected hydrazides in flow.^61^ The custom-made photochemical reactor consists of a perfluoroalkoxy capillary reactor (PFA, 750 μm ID, 5 mL volume) cooled by a fan, which is irradiated with six dimmable high-intensity UV-A chip-on-board LEDs, providing a maximum of 144 W optical power. When a CH_3_CN/HCl_0.1M_ 7 : 1 solution containing 8.1 and 8.2 was irradiated (*λ* = 365 nm, 144 W) in the presence of TBADT (0.2 mol%), compound 8.3 was isolated in 73% yield (corresponding to 12 mmol h^−1^). With this new powerful technology in hand, the authors pushed the productivity of 8.3 up to 2.15 kg per day by simply adjusting flow rates and the capillary length. In addition, telescoped approaches were developed for the synthesis of pyrazoles (8.4) and phthalazinones (8.5). Overall, the flow approach allowed to improve the efficiency of the reaction compared to batch alternatives, enabling both small scale (1 mmol) and pilot scale (>1 kg) operation in a single photochemical microreactor.

**Fig. 9:**
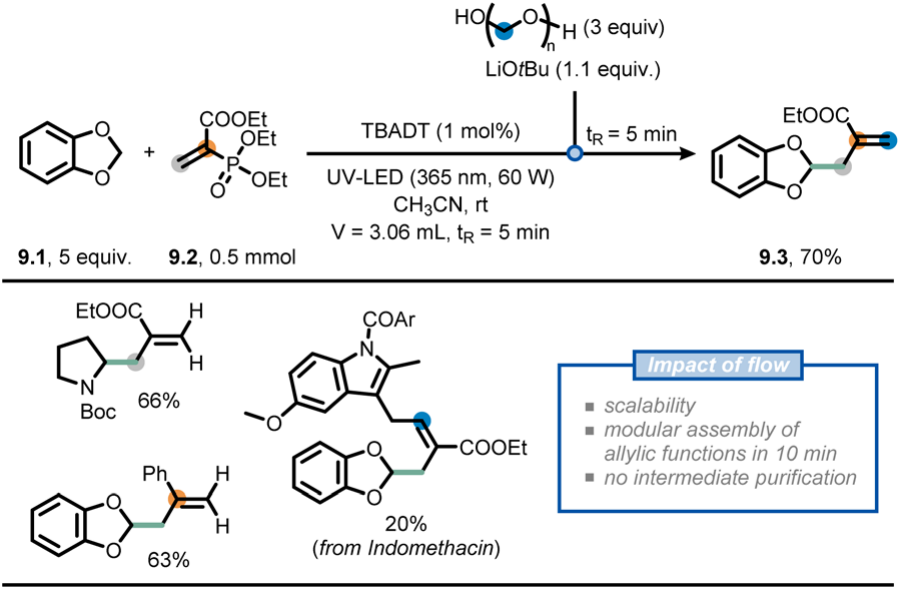
Use of flow chemistry to devise a flexible approach for the installation of allyl moieties at strong aliphatic C(sp^3^)–H bonds.

Another recent example involves the modular allylation of unactivated C(sp^3^)–H bonds *via* a telescoped approach in flow (Fig. 9).^62^ Hereto, the authors merged decatungstate photocatalysis with classical Horner–Wadsworth–Emmons (HWE) olefination chemistry, exploiting the intrinsic modularity of flow chemistry, to append hydroalkanes with an allyl functional group. First, an acetonitrile solution of acrylate 9.2, benzodioxole (9.1) and decatungstate as the photocatalyst was injected into a Vapourtec UV-150 photochemical reactor (*t*_R_ = 5 min) equipped with a 60 W UV-A LED light source (*λ* = 365 nm) to deliver the corresponding Giese adduct. In contrast, when the same reaction was conducted in batch, full conversion was reached only after 20 hours. Second, the stream containing the Giese adduct was subsequently merged with a stream containing LiO*t*Bu and paraformaldehyde to perform a HWE olefination. By doing so, the allylated product 9.3 was isolated in an overall 70% yield, requiring no intermediate purification. The developed two-step flow process could be subsequently extended to a wide variety of aliphatic and aromatic aldehydes, as well as deuterated paraformaldehyde, to prepare various highly functionalized allylated compounds. These moieties are difficult to prepare *via* conventional radical allylation approaches.^63,64^

**Fig. 10:**
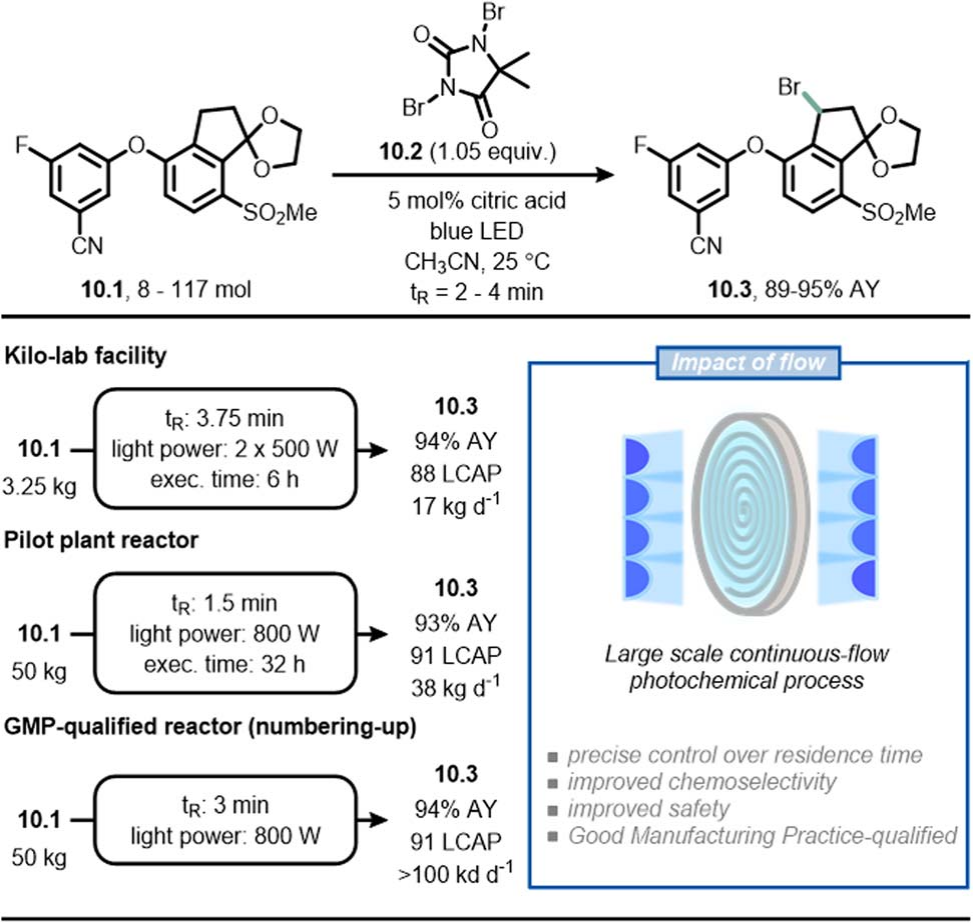
Adoption of flow chemistry in an industrial setting to improve chemoselectivity and facilitate scalability in the synthesis of 10.3, an intermediate *en route* to belzutifan.

As a final example, scientists at Merck have reported a scalable continuous-flow photochemical process for the bromination of intermediate 10.1, which is required to produce belzutifan (Fig. 10), a drug for the treatment of renal cell carcinoma.^65,66^ In the traditional synthetic route, the radical benzylic bromination was achieved through the use of azobisisobutyronitrile (AIBN) as initiator and reagent 10.2 as the bromine source. Under these reaction conditions, 10.1 is relatively unstable and undergoes sudden di-bromination and de-ketalization. By replacing AIBN with blue light, the authors found that the reaction could be conveniently stopped to get the product in 91% LCAP (Liquid Chromatography Area Percent) in batch in only 3 minutes. The scale up of the process occurred gradually in three steps using flow technology. In the first step, 3.5 kg of 10.1 was converted to 10.3 in 88 LCAP and 94% assay yield with a residence time of 3.75 minutes; in the second step, 50 kg of 10.1 was converted to 10.3 in 91 LCAP and 93% assay yield with a residence time of 1.5 minutes. Finally, the researchers adopted a numbering-up approach to deliver more than 100 kg per day (91 LCAP and 94% assay yield) of the product. In this case, the synthetic advantage offered by flow chemistry is the improved chemoselectivity because it enabled a precise control over reaction time, which prevented undesired reactions such as overbromination of 10.1.

## 6. Electrochemistry

Another field that has benefited significantly from the adoption of microfluidic technologies is electrochemistry.^67–71^ Electrochemical reactions are by definition heterogeneous processes, as they rely on a redox event between an electrode surface and a molecule in solution. Accordingly, mass transfer from the bulk to the electrode surface becomes a very important consideration. However, using microfluidic setups, the impact of poor mass transfer can be minimized due to the larger surface-to-volume ratio. Notably, the small interelectrode distances in microreactors reduces the observed ohmic voltage drop, enabling a net reduction of the amount of supporting electrolyte needed in the reaction mixture.

**Fig. 11:**
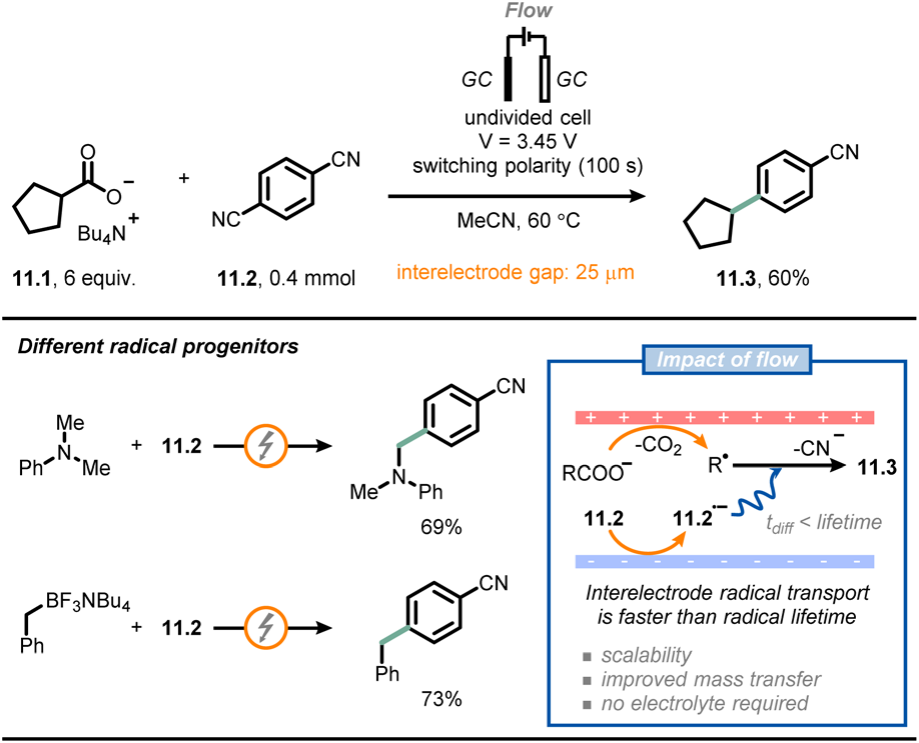
Microfluidic electrochemical cells with short inter-electrode gaps enable redox-neutral transformations in the absence of supporting electrolytes. GC: glassy carbon.

A collaborative effort between Buchwald, Jensen *et al.* resulted in the development of a μRN-eChem (microfluidic redox-neutral electrochemical) cell to perform redox-neutral transformations (Fig. 11).^72^ Although these transformations could in principle be run under photoredox catalytic conditions, the use of photocatalysts might bring around some inconveniences, such as the challenging tuning of redox potentials, the use of expensive transition metals and the instability of photocatalysts under operating conditions. The flow cell consists of two laser-micromachined glassy carbon electrodes separated by a FEP gasket with a reduced thickness (25 μm). Due to the short inter-electrode distance, it is possible to reduce the time required for interelectrode migration (as governed by the following equation: *t* = *d*^2^/*D*; *d*: interelectrode distance, *D*: molecular diffusivity) significantly, so that it is shorter than the lifetime of open-shell intermediates generated upon electrolysis, which paves the way to efficient redox-neutral manifolds. For example, the authors coupled Kolbe electrolysis with arene reduction to develop a decarboxylative arylation by capitalizing on the persistent radical effect.^73^ Thus, arene 11.2 is reduced at the cathode generating a persistent radical anion 11.2^˙−^; the latter radical intermediate migrates towards the anode, where Kolbe electrolysis yields transient alkyl radicals. Subsequent radical–radical anion coupling occurs to afford the arylated product 11.3 after decyanation. Intriguingly, the small inter-electrode gap also reduces the ohmic drop, thus eliminating the need for additional supporting electrolyte. A similar approach could be used for different alkyl radical progenitors (*e.g.*, arylamines or trifluoroborate salts).

**Fig. 12:**
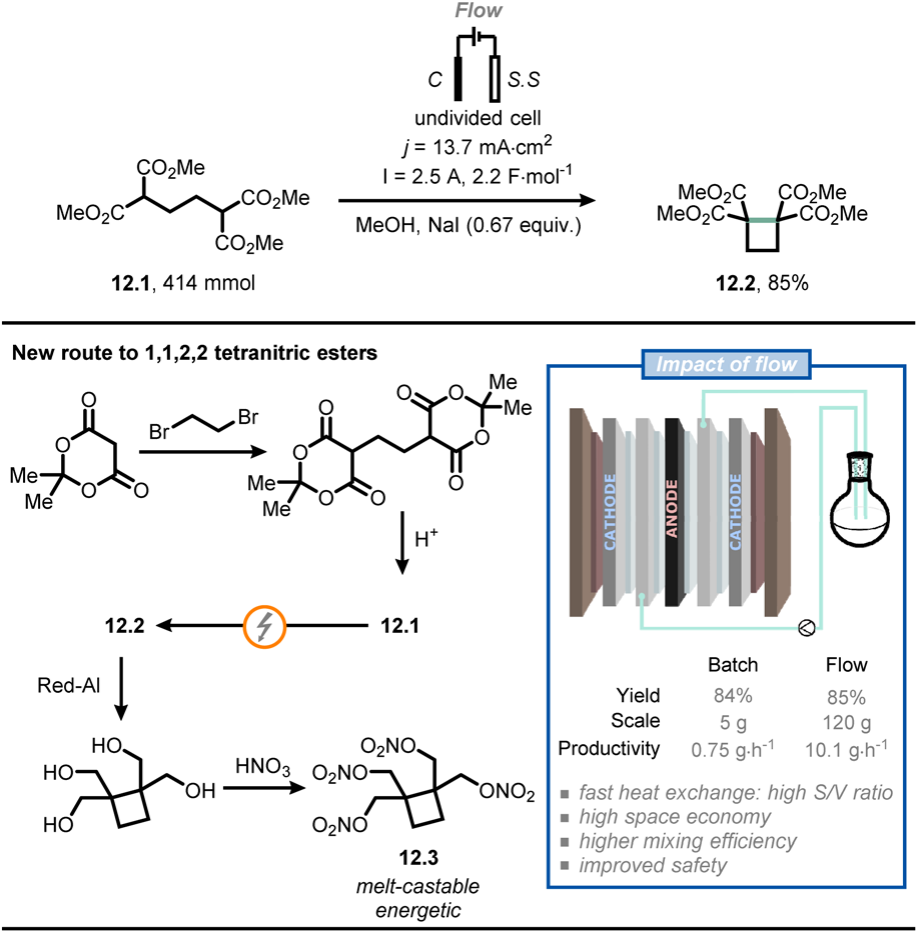
Flow electrochemistry facilitates the safe scale-up of a promising melt-castable energetic intermediate. C: graphite electrode; S. S: stainless steel electrode.

Baran and co-workers used a flow electrochemical approach to synthesize compound 12.2, an intermediate for the synthesis of cyclobutane-containing tetranitric esters (Fig. 12).^74^ One of these compounds, namely cyclobutane-1,1,2,2-tetrayltetrakis(methylene) tetranitrate (12.3), proved to be valuable as a potential melt-castable energetic. As the original route for the synthesis of 12.2 posed several concerns in terms of safety and expenses,^75^ the authors were eager to find a more practical alternative. Compound 12.1 was synthesized starting from Meldrum's acid followed by acidic hydrolysis; next, electrolytic conditions were adopted to promote cyclization to give compound 12.2. In the original route, the latter step was conducted in batch and required the use of expensive electrodes (*e.g.*, Pt) on a relatively small scale (gram scale). However, the authors managed to translate the reaction to flow on a 120 g scale by using cheap electrodes: a graphite anode and two stainless steel cathodes, connected in a monopolar fashion. Full electrolysis was reached in 10 hours under galvanostatic conditions at 2.5 A, delivering *ca.* 100 g of 12.2 (85% yield after isolation), which corresponds to a productivity of 10.1 g h^−1^. In comparison, when the reaction was run in batch on a 5 g-scale, a productivity of 0.75 g h^−1^ was achieved. Finally, compound 12.2 was converted to 12.3*via* reduction by Red-Al and esterification. Overall, flow chemistry enabled in this specific case a straightforward and safe scale up of a value-added intermediate (12.2), which should facilitate the development of a safe industrial process of 12.3.

Flow electrochemistry is also extremely appealing to the chemical industry due to the ability to use “green” electricity derived from solar and wind energy. Furthermore, the reduced inter-electrode gap introduces a series of advantages that cut the costs of conducting electrochemical reactions. Indeed, voltages needed to promote reactivity are generally lower (due to a reduced overpotential), which also allows to reduce the amount of expensive supporting electrolyte and thus facilitates the purification processes. Flow electrochemistry is also safer compared to batch because it enables a tighter heat management control, avoiding thermal runaways,^31^ and due to its continuous nature, it also prevents occurrence of dead zones (*i.e.*, regions in a reactor where the reaction mixture is stagnant).

**Fig. 13:**
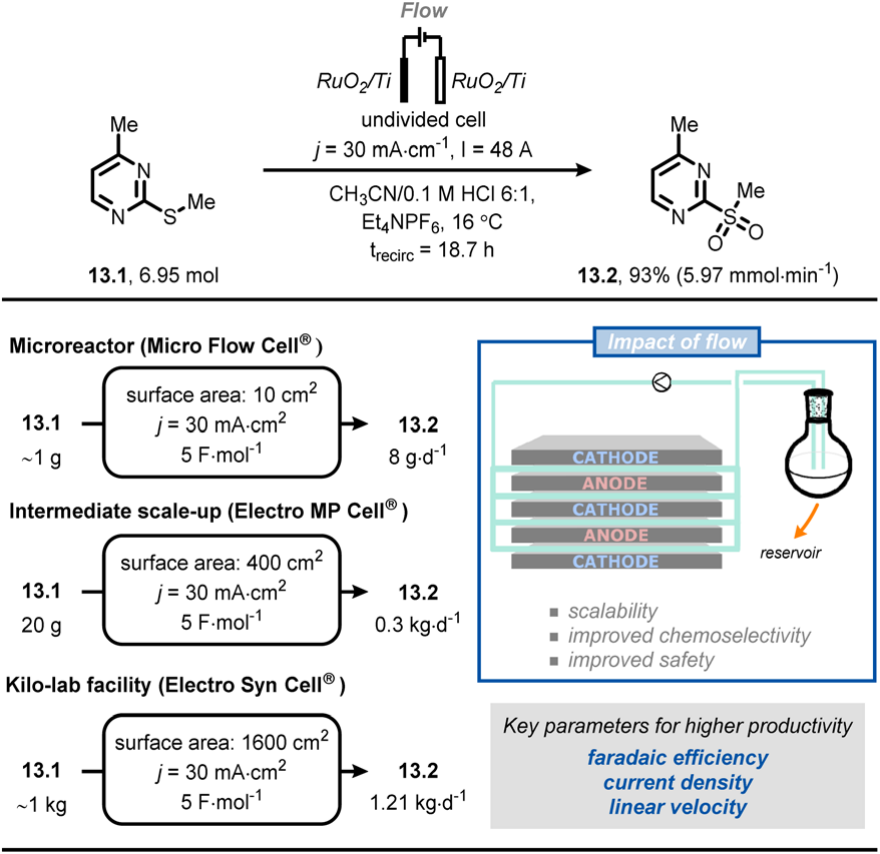
Adoption of flow electrochemistry in an industrial context for the highly scalable, selective anodic oxidation of thioether 13.1. *t*_recirc_: time of recirculation.

Very recently, scientists at Merck developed a scalable flow process for the selective anodic oxidation of thioethers.^76^ In this process, thioether (13.1) was oxidized to the corresponding sulfone (13.2) (Fig. 13). Compound 13.1 is a fragment of a drug candidate but its chemical oxidation with classical oxidants (*e.g.*, oxone, *m*-CPBA, periodate or tungstate) led to a complex mixture of the corresponding sulfoxide and sulfone. After a brief reaction optimization in batch, the authors successfully translated their protocol into flow by exploiting a recirculating mode operation. It was possible to gradually scale the process from gram-to kilogram-scale by systematically increasing the electrode surface area, whilst keeping the current density and electron equivalents constant. Ultimately, it was possible to prepare 1.11 kg of 13.2 using 1600 cm^2^ electrodes and applying a current density of 30 mA cm^−2^, a current of 48 A, 4.5 F mol^−1^, for *ca.* 19 h of processing time, thus meeting the criteria for pilot production (1.21 kg per day).

## 7. Safety

In addition to fast heat and mass transfer of microreactor technology, the overall safety of the process is greatly improved due to the small reactor volumes and precise control of reaction conditions.^32,77,78^ Hazardous reactions or even reactions that are impossible in conventional conditions can be carried out with relatively low risk in flow conditions, such as processes involving toxic, reactive gases and explosive reagents.

**Fig. 14:**
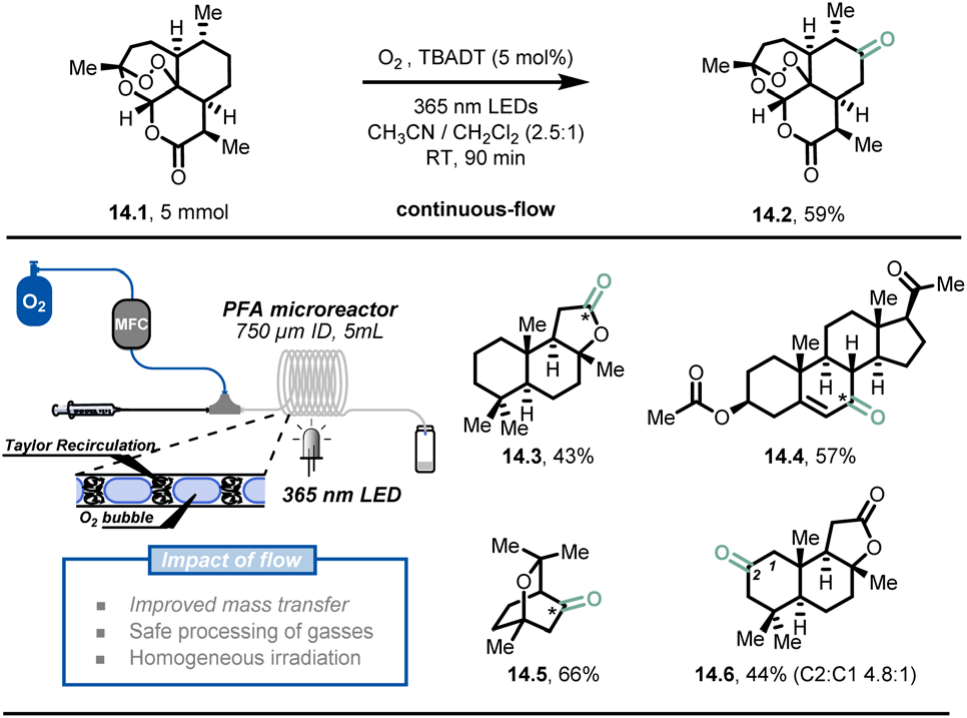
Decatungstate-mediated C(sp^3^)–H oxidation with oxygen in continuous flow. TBADT: Tetra-*n*-butylammonium decatungstate. Reproduced from ref. 81 with the permission of Wiley-VCH, copyright 2018.

It is understandable that the use of toxic and dangerous gases is highly restricted in modern synthetic laboratories. In order to perform gas-based transformations and achieve decent results, dedicated high-pressure gas reactors with multiple detectors are required. This is why the direct use of molecular oxygen^79,80^ as a simple, green oxidant is discouraged in conventional batch systems. However, Noël *et al.* developed a simple, selective, and safe decatungstate-photocatalytic aerobic oxidation of C(sp^3^)–H bonds under flow conditions (Fig. 14).^81^ The oxygen stream was delivered by a mass flow controller (MFC) and merged with the liquid stream infused by a syringe pump, delivering a uniform segmented flow regime in the continuous-flow photoreactor (PFA tubing, 750 μm I.D.). A typical interfacial area from 3400 to 9000 m^2^ m^−3^ could be obtained through Taylor recirculation patterns,^82^ leading to an ideal mixing between the oxygen and the liquid solution. Within 90 minutes of residence time and 5 mol% TBADT loading, the authors demonstrated that artemisinin (14.1) could be converted to its natural derivative artemisitone-9 in 59% yield (14.2, 5 mmol scale) with this methodology. Furthermore, most of the activated and unactivated aliphatic bonds (30 examples) such as (−)-ambroxide 14.3, pregnenolone acetate 14.4, eucalyptol 14.5 and (+)-sclareolide 14.6, could be selectively oxidized in moderate to excellent yield (43–91%).

**Fig. 15:**
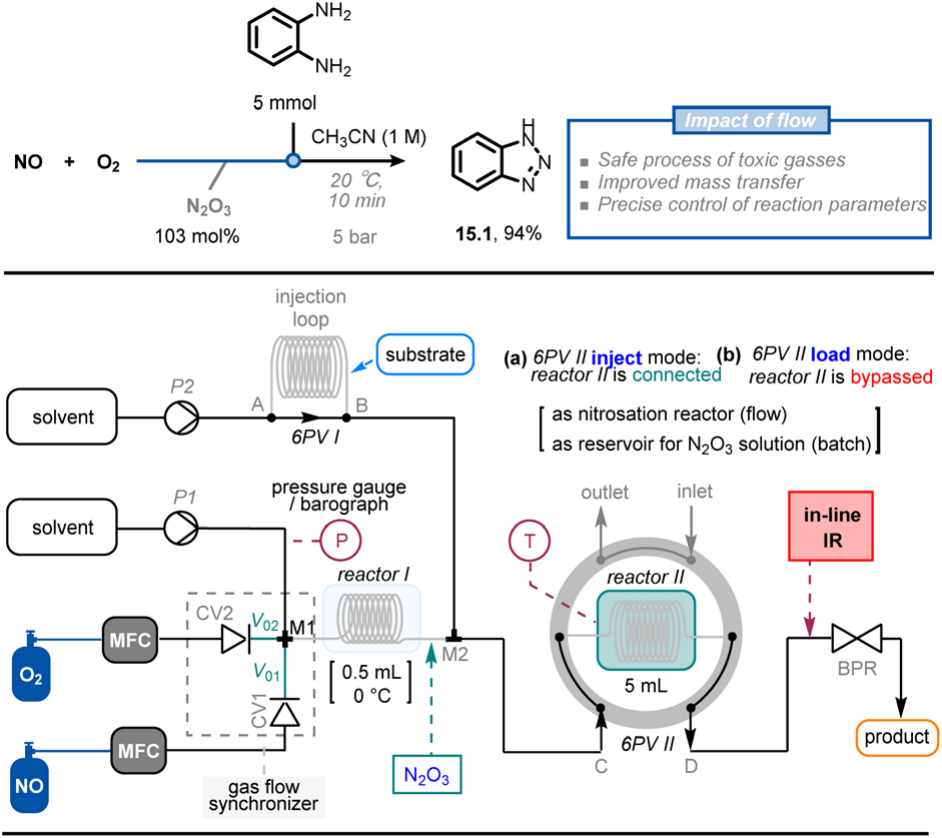
Continuous-flow setup for the generation of N_2_O_3_ and its subsequent use in synthetically-relevant contexts. CV: check valve; 6PV: six-position switch valve. MFC: Mass-Flow Controller. Reproduced from ref. 83 with the permission of Wiley-VCH, copyright 2022.

In addition to safely handling toxic reagents in flow, microreactor technology allows for the generation and utilization of sensitive reaction intermediates without the need to store hazardous quantities of materials. For example, dinitrogen trioxide (N_2_O_3_) is a powerful nitrosating reagent with an appealing atom economy, but it is only stable under cryogenic conditions and in a NO atmosphere. Furthermore, nitrosation with pure N_2_O_3_ is fast and highly exothermic, which limits its use both in laboratories and industry. Recently, Monbaliu *et al.* developed a continuous-flow methodology for the synthesis and use of anhydrous N_2_O_3_ solution, minimizing the safety concerns associated with this toxic gas and exothermic reactions (Fig. 15).^83^ The anhydrous N_2_O_3_ solution (up to 1 M) was generated in a flow setup using NO and O_2_ streams as starting materials. A telescoped method was then developed to synthesize two types of *N*-heterocycles (*i.e.*, benzotriazoles 15.1 and 3-substituted sydnones, >30 examples) in moderate to excellent yield (54–99%).

**Fig. 16:**
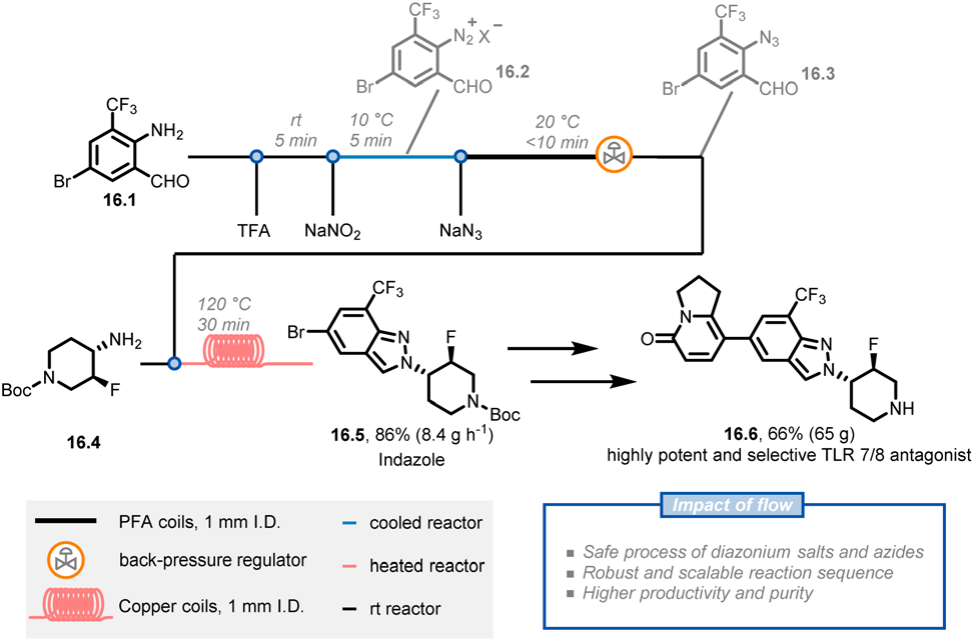
Continuous flow synthesis of indazole 16.5, involving a three-step synthetic sequence containing diazonium salts and azide generation. TFA: trifluoroacetic acid.

Diazonium salts, which are typically unstable and generate large volumes of nitrogen gas during synthetic transformations, can be problematic to scale up in batch systems and raise many safety concerns. Chemists at Novartis have reported a scalable and multi-step continuous-flow procedure for synthesizing 2*H*-indazoles in which hazardous diazonium salt and azide chemistries are safely handled on a 200 gram scale (Fig. 16).^84^ The key intermediate, 2*H*-indazole 16.5, is used to synthesize compound 16.6, a highly potent and selective inhibitor for the treatment of autoimmune disorders. The researchers initiated the synthetic route with amino-aldehyde 16.1 and applied a diazotization and azidation protocol using sodium nitrite with trifluoroacetic acid followed by a reaction with sodium azide at a lower temperature to obtain azide 16.3. Instead of isolating the latter compound, the researchers obtained a 0.3 M solution of azide in dichloroethane (91% yield and 14.1 g h^−1^ productivity). After further concentrating the azide solution to 0.45 M, a cyclization reaction was conducted with amine 16.4 through two 10 mL copper coils to afford 217 g of indazole 16.5 in 94% purity and 95% yield (86% total yield). 16.5 was then used without additional purification in the subsequent steps to obtain the target compound 16.6 in high quality and acceptable yield. Notably, the continuous-flow procedures were suitable for scale up with only minor modifications, affording 16.5 on a kilogram scale.

## 8. Scalability

The use of flow chemistry in large-scale production has several advantages over traditional batch chemistry, among which an easier adaptation of laboratory-scale reactions to larger production scales without sacrificing reaction performance.^85,86^ Two approaches can be followed for scaling up flow chemistry reactions: numbering up and sizing up.^54,87^ Numbering up involves increasing the number of channels in the microreactor, while sizing up involves increasing the length and/or diameter of the channels. These strategies can be described mathematically by the equation of the volume of the microreactor, where *V*, *N*, *L*, and *D* represent the volume of the reactor, the number of channels, the length of the channels, and the diameter of the channels, respectively.^87^
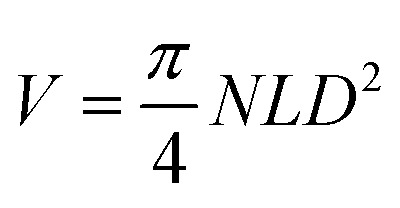


**Fig. 17:**
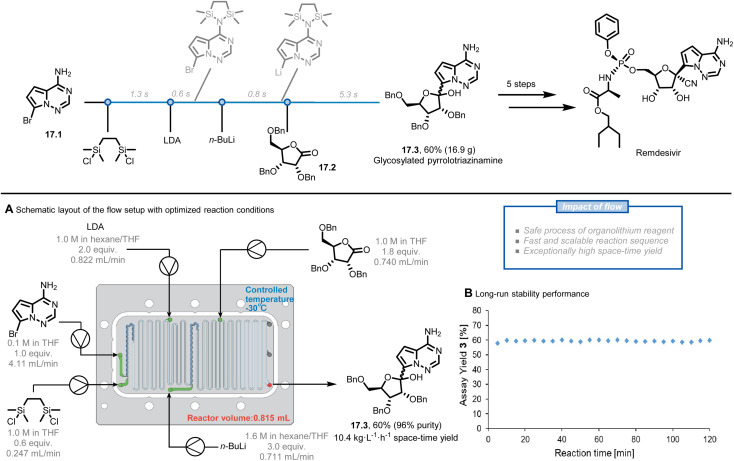
Continuous-flow process to generate remdesivir intermediates. LDA: lithium diisopropylamide. Reproduced from ref. 90 with the permission of American Chemical Society, copyright 2021.

Remdesivir, the first COVID-19 drug approved by the U.S. FDA, has been highly sought after due to the rapid spread of the COVID-19 pandemic. The synthetic pathway towards Remdesivir starts with the C-glycosylation of the halogenated pyrrolotriazinamine 17.1 with benzyl-protected d-ribonolactone 17.2 to obtain the key intermediate 17.3, which was identified as an obstacle for large-scale production.^88,89^ The reported organometallic step using organolithium reagents generally requires long addition periods and cryogenic temperatures due to their fast and exothermic characteristic, while benefiting the most from the enhanced mass and heat transfer of the flow system. Kappe *et al.* reported a five-step continuous flow process, facilitating this highly exothermic C-glycosylation (Fig. 17A).^90^ Before translating the reported reaction conditions to flow, they repeated the reaction in batch to rule out the potential issues, such as the formation of solids directly after the addition of 1,2-bis(chlorodimethylsilyl)ethane (BCDSE) in the first step. The precipitation was assumed to be the protonated by-product of heterocycle 17.1 and its formation would lead to microreactor clogging. The solid was indeed observed within approximately 5 s at ambient temperature, leaving only a narrow window to add a base for scavenging HCl. Such a short residence time can be easily achieved in flow. Through a careful analysis of the reaction sequence and precise control of the process parameters, the authors achieved a 60% yield of glycosylated product 17.3 at a moderate temperature (−30 °C) in a total residence time of only 8 s. A stable and scalable process was further demonstrated for 2 h (Fig. 17B), providing a throughput of 8.5 g h^−1^ (10.4 kg L^−1^ h^−1^ space-time yield) within a small reactor volume of only 0.815 mL. These results present the potential to reach even higher production of the key glycosylated intermediate 17.3 and remdesivir exploiting the general scale-up strategies in flow, such as numbering up.

**Fig. 18:**
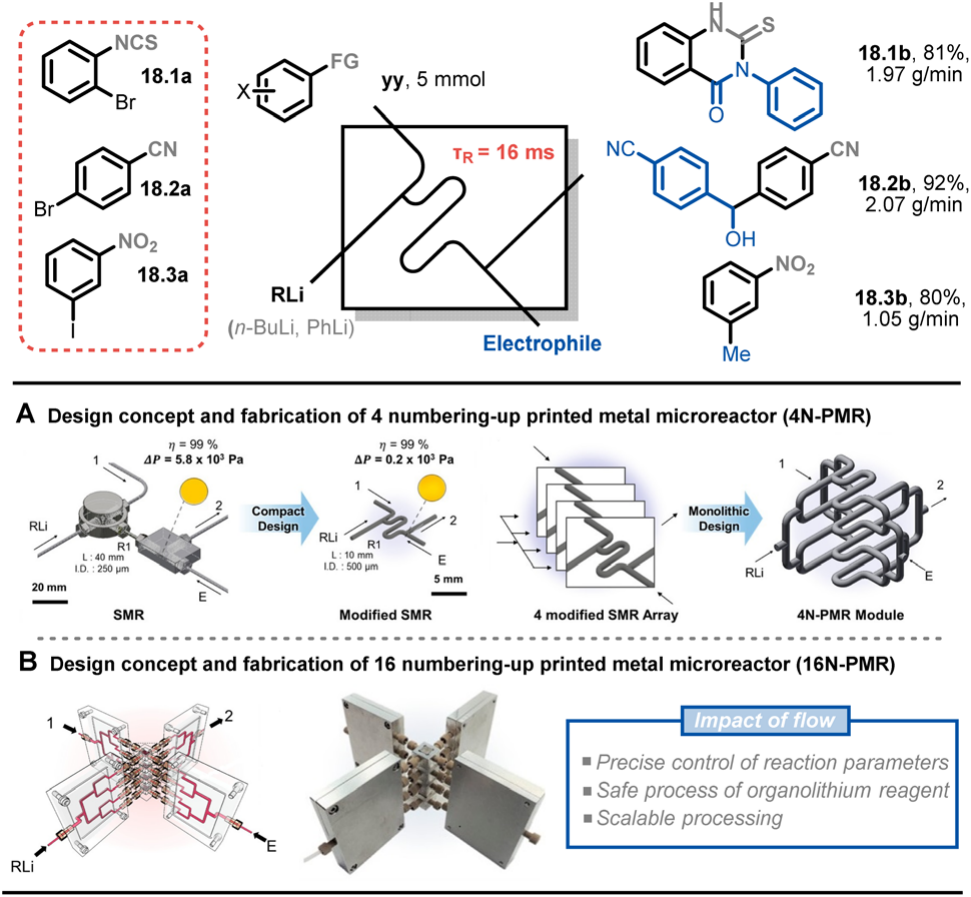
Scale-up strategy for an ultrafast sub-second flash chemistry exploiting aryllithium intermediates using numbered-up 3D-printed microreactors. Reproduced from ref. 91 with the permission of American Chemical Society, copyright 2022.

Numbering up is a common strategy in the scale-up of flow chemistry because it enables the retention of the hydrodynamics and transfer properties associated with the micro-environment (*e.g.*, mixing, heat transfer, irradiation efficiency). This allows reactions to occur under conditions that are identical to those in an individual microreactor. Kim and co-workers presented a scale-up process of ultrafast sub-second chemistry to synthesize drug scaffolds *via* 16 numbered-up printed metal microreactors (16N-PMR).^91^ As previously mentioned, reactions with organolithium reagents are fast and highly exothermic. To precisely control the lithiated intermediates, an optimal residence time of around 16 ms with a flow rate of 7.5 mL min^−1^ was used, which created a significant pressure drop in the microreactor. To address this issue, the authors developed a modified microreactor with a larger circular channel diameter, reducing the pressure drop by 28 times under the same flow conditions (Fig. 18A). After simulating the maldistribution factor of the internal bifurcation distributor (<1%), a monolithic 4N-PMR with a validated structure was fabricated, which was further demonstrated with ultrafast chemistry, giving yields of 84–99% of 12 products. To further scale up this type of chemistry, a 16N-PMR was assembled by connecting four 4N-PMRs and four external flow distributors with 1/8-inch tubes (Fig. 18B). The 16N-PMR module exhibited a high output efficiency of 1–2 g min^−1^ (approximately 3 kg per day) of three drug scaffolds 18.1b–18.3b.

**Fig. 19:**
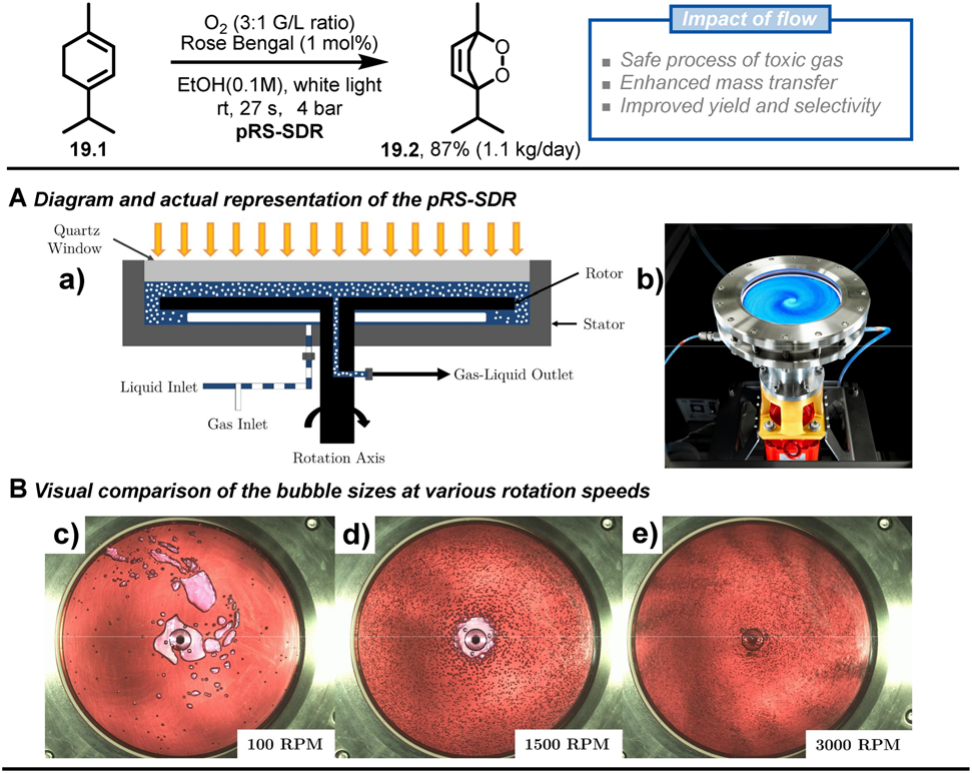
Scale up of the [4 + 2] cycloaddition between terpinene and photochemically generated singlet oxygen using rotor-stator spinning disk reactor technology. Reproduced from ref. 93 with the permission of Elsevier, copyright 2020.

Besides passive microreactors (which exploit the flow energy delivered by the pumps to induce mixing), active microreactors that utilize external energy are also commonly employed, particularly for scaling heterogeneous chemistry up. Multiple types of active continuous reactors have been reported, such as rotating disk reactors,^92–94^ oscillatory flow reactors,^95–97^ thin-film rotating reactors,^98^ CSTR^99,100^ and ultrasonic reactors.^101,102^ To demonstrate the advantages of such flow chemistry technology, Van der Schaaf and Noël *et al.* reported a high-throughput photochemical rotor-stator spinning disk reactor (pRS-SDR) to enhance the gas–liquid mass transfer and perform a large-scale gas–liquid photooxygenation of α-terpinene (19.1).^93^ A previous report^103,104^ and some preliminary experiments in batch and flow (which increased selectivity from 50% to 70%) indicated that better light irradiation and mass transfer were critical to this transformation, which suffers from deleterious side reactions due to overoxidation and triplet oxygen-enabled oxidations. Furthermore, scaling up gas–liquid oxidation chemistry in batch systems is challenging due to poor gas dispersion and the potential for hazardous situations related to the oxygen-rich environment in the headspace. The developed pRS-SDR enables efficient dispersion and rapid mixing of the gas–liquid phases by the fast-rotating disk between two stators positioned at a short distance (1–2 mm) (Fig. 19A), resulting in an enhanced mass transfer rate,^105^ which is beneficial for gas–liquid transformations. The reaction solution was irradiated from a quartz window by a 120 W white LED light source, affording an illuminated volume of 27 mL. After investigating all the reaction parameters, such as gas/liquid ratio and rotational speed, a productivity of 1.1 kg per day of ascaridole 19.2 (87% yield and 90% selectivity) was reached with a residence time of only 27 s, which convincingly proved that the enhanced mass transfer rates obtained in the pRS-SDR are crucial to achieving excellent multiphase transformations.

## 9. High-throughput experimentation and automation

**Fig. 20:**
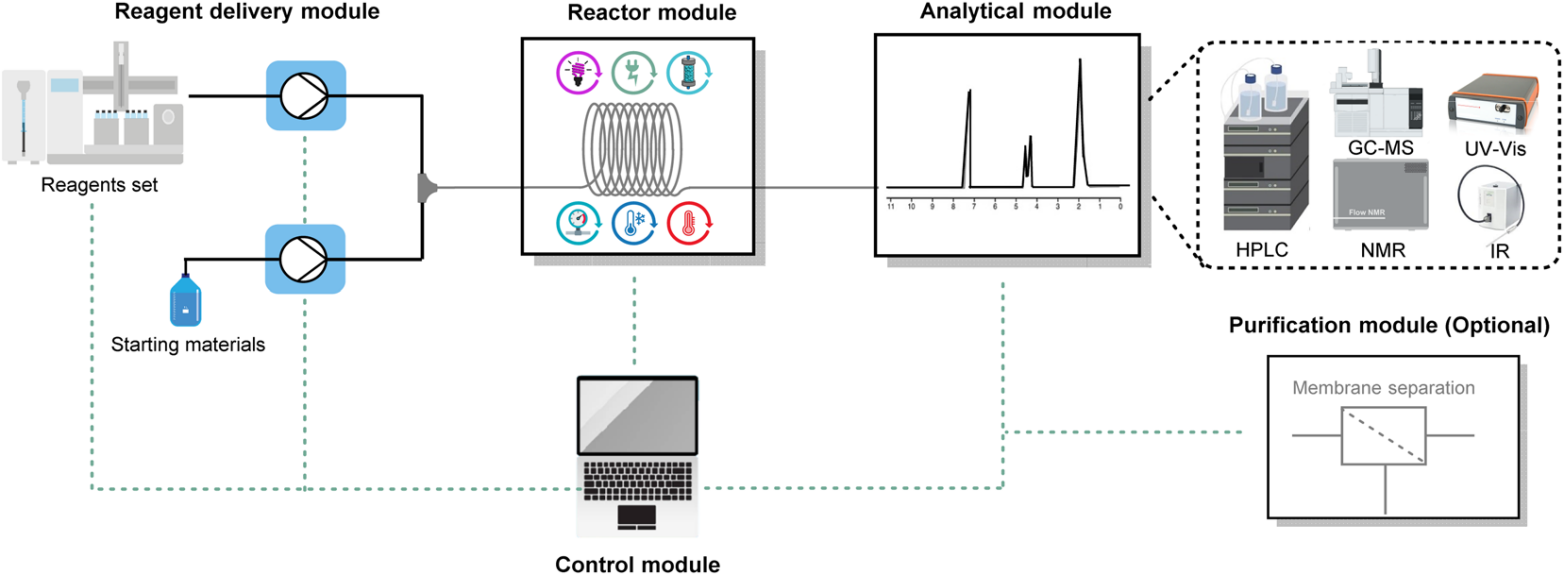
General configuration of an automated and modular continuous-flow platform.

By integrating high-throughput experimentation (HTE)^106–109^ with process analytical technology (PAT),^110–113^ optimization of continuous-flow reactions can be done efficiently and with a wealth of data (Fig. 20). This integration results in fast data acquisition, closed-loop experimentation,^114,115^ and improved discovery and reproducibility of organic synthesis in flow, leading to data-driven or algorithm-driven autonomous experimentation.^116,117^

Batch-based HTE^106,108,109,118,119^ suffers from some limitations, such as a lack of pressure and temperature screening, the need for non-volatile solvents, and the potential for cross-contamination. In contrast, Pfizer researchers showed that flow-based HTE with two LC/MS instruments could be effectively handled to screen thousands of Pd-catalyzed Suzuki–Miyaura couplings under high pressure and temperature conditions, by simply adding a back pressure regulator, allowing to produce a large dataset of 5760 reactions at a rate of over 1500 samples per day.^120^

**Fig. 21:**
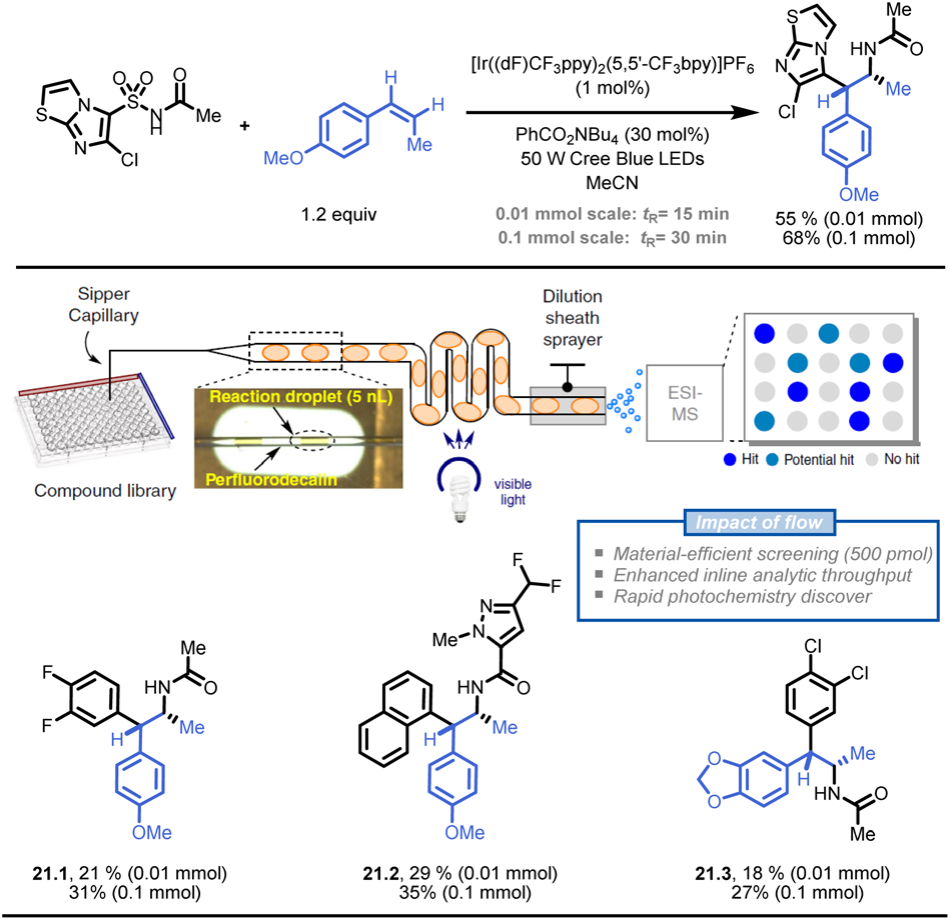
Development of a droplet-based flow-HTE platform to prepare API libraries in a time- and resource-efficient fashion. Reproduced from ref. 121 with the permission of Springer Nature, copyright 2020.

Stephenson and colleagues also developed a droplet-based microfluidic platform for flow-based HTE to generate pharmaceutically relevant compound libraries (Fig. 21).^121^ The platform utilized an oscillatory flow photoreactor and electrospray ionization-mass spectrometry (ESI-MS) to analyse the reactions. With a throughput of 0.3 samples per second, ESI-MS detected 37 hit conditions, and seven of nine selected reactions were successfully validated through product isolation. This droplet-based method can quickly discover photochemical reactions and generate compound libraries in flow (*e.g.*, 21.1–21.3).

More researchers have been exploring automated telescoped multistep flow synthesis,^111,122–128^ with a common approach being to combine modular unit devices in a linear sequence, known as “Lego-like synthesis”.^122,129^ The integration of process analytical technology (PAT) tools and advanced data analysis models can further improve reaction understanding and acceleration of the optimization rate.^111^ There have also been reports of unique approaches, such as a radical synthesizer with a central hub and access to individual reactors, enabling the storage of chemical intermediates and removing limitations from previous steps.^128^ Using this strategy, several analogues of rufinamide were prepared without the need for physical reconfiguration of the system.

**Fig. 22:**
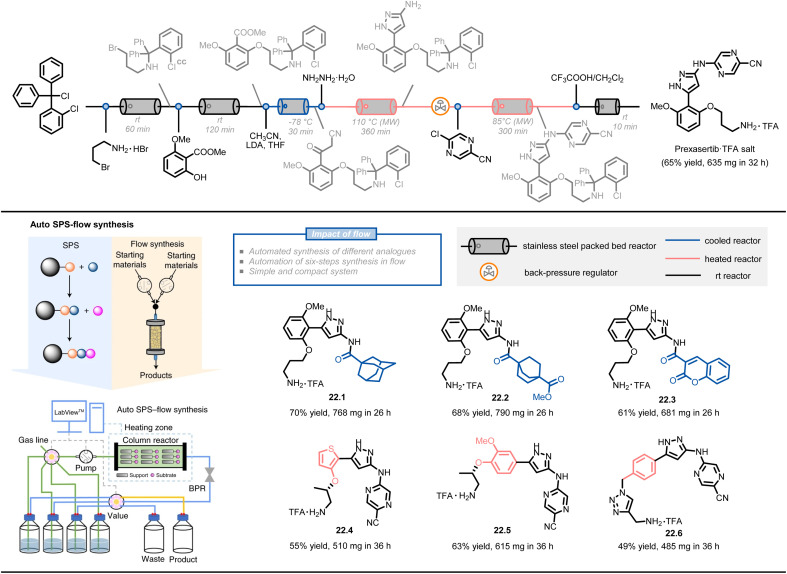
Schematic representation of an automated SPS-flow synthesis of prexasertib and derivatives. LDA: lithium diisopropylamide. Reproduced from ref. 124 with the permission of Springer Nature, copyright 2021.

Another approach combines solid-phase synthesis and continuous-flow chemistry to perform automated multistep synthesis of active pharmaceutical ingredients (APIs) (Fig. 22)^124^. In this method, starting materials are covalently attached to a solid support and undergo growth through sequential treatment with different reagents, avoiding intermediate isolation and compatibility issues. A compact system design, including multi-position selection valves, pumps, and a stainless-steel column reactor, was able to perform a six-step synthesis of prexasertib with a 65% isolated yield in 32 hours of continuous execution. The procedures were converted to a computerized chemical recipe file (CRF) and successfully used for the synthesis of 23 prexasertib analogues with moderate to good yields (*e.g.*, 22.1–22.6, 49–70%).

Besides the use of human intervention to refine the automated continuous-flow platforms, algorithm-driven optimization has gained significant attention in both academia and industry as a way to explore high-dimensional chemical space and achieve optimal conditions with fewer experiments.^107,130–136^ There are three main types of algorithms applied for self-optimization experimentation: local optimization algorithms such as design of experiments (DoE)^137,138^ and Nelder–Mead simplex,^139,140^ global optimization algorithms like SNOBFIT^141,142^ and Bayesian Optimizations,^114,130,143–145^ and machine learning algorithms like deep reinforcement learning.^146^

**Fig. 23:**
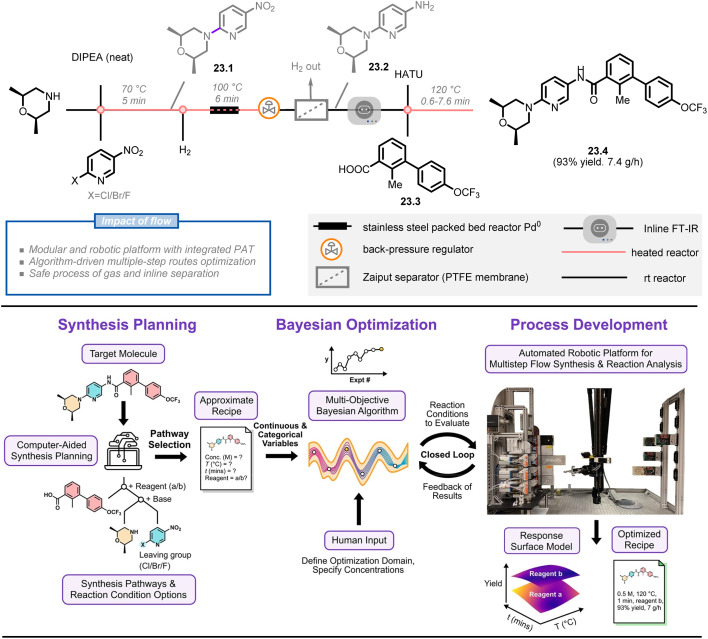
Overall approach for bayesian optimization algorithm-driven multistep-synthesis of Sonidegib on an automated robotic platform. DIPEA: *N*,*N*-diisopropylethylamine, HATU: hexafluorophosphate azabenzotriazole tetramethyl uronium. Reproduced from ref. 150 with the permission of American Chemical Society, copyright 2022.

Jensen *et al.* are pioneers in this growing field, having developed various versions of automated continuous-flow platforms, including a fridge-size reconfigurable platform,^147^ a ‘plug-and-play’ platform,^142^ and a robotic platform.^148^ Their most recent development is a bayesian optimization-driven automated robotic flow platform that includes computer-aided synthesis planning (CASP),^149^ multi-objective optimization and robotic-enhanced multistep synthesis (Fig. 23).^150^ To demonstrate the power of this platform, the authors selected a high-ranked synthetic pathway for the molecule sonidegib 23.4 using open-source CASP software (ASKCOS) and manual assessment of synthetic feasibility. This pathway was then optimized on a modular platform that included a fast-moving 4-axis gantry robot, two types of reactors (heated and packed bed), and three analytical modules (inline FT-IR, LC-MS, HPLC). Five optimization variables (two categorical parameters, *i.e.* activation reagent and coupling reactor volume, and three continuous parameters, *i.e.* activation time, 23.1 : 23.3 ratio and coupling temperature) and two objective functions (yield and productivity of sonidegib) were considered in a multiple-step campaign using the bayesian optimization algorithm. In just 15 total experiments over a 13 hours period (8 initialization and 7 refinement runs), the algorithm identified the optimal conditions with simultaneous high yields and productivities of the product (93% yield, 7.4 g h^−1^), showcasing the potential for machine learning, automation, and robotics to enhance manual experimentation.

Finally, the integration of automated and flow-based high-throughput experimentation (HTE) platforms enables fast and large-scale data generation, opening the door to data-driven techniques^151–154^ for discovering valuable reactivity insights and exploring novel synthesis strategies. Contributing to the open reaction database^155^ or following the FAIR principles^156^ in data sharing will further facilitate the integration of data science in organic synthesis. As the field continues to evolve, it holds significant potential for closed-loop experimentation and full autonomy in synthesis operations.

## 10. Conclusions

Synthetic chemistry has been a major player in discovering new medicines, materials, and fine chemicals. While the primary focus of synthetic chemists has always been on the development of new reactivity concepts, the reactor has systematically been overlooked by the community. However, as shown in this review, flow chemistry is packed with perks that can push the boundaries and unleash unique reactivity and selectivity in synthetic organic chemistry. Flow reactors not only make new synthetic routes a reality, they fast-track them from lab to large scale production. And, as flow chemists master both chemical and engineering principles, they become valuable links between lab discovery and production engineering.

With this review showcasing how flow chemistry can team up with methodological development, we aimed to provide a helpful field guide for those eager to dive in. And with the continued growth of interest in flow chemistry, we expect even more exploration and optimization of synthetic organic chemistry. We believe flow technology will become a must-have in every chemical lab and be just as familiar as the classic round-bottom flask.

## Data availability

The most common hurdles to enter the field of flow chemistry and an overview of essential accessories are provided in the ESI.†

## Author contributions

L. C. and Z. W. wrote the review and contributed equally. The concept and the editing of this review was done by T. N.

## Conflicts of interest

There are no conflicts to declare.

## Supplementary Material

SC-014-D3SC00992K-s001
