# Supplementary material for: A field guide to flow chemistry for synthetic organic chemists
Source: Chem Sci. 2023 Mar 15;14(16):4230–47. doi: 10.1039/d3sc00992k (PMC10132167; doi:10.1039/d3sc00992k)

Electronic Supplementary Material (ESI) for Chemical Science.
This journal is © The Royal Society of Chemistry 2023

Supporting Information

A field guide to flow chemistry for synthetic organic chemists

Luca Capaldo,[†a] Zhenghui Wen[†a] and Timothy Noël[*a]

[a] Flow Chemistry Group, van 't Hoff Institute for Molecular Sciences (HIMS), University of Amsterdam, 1098 XH Amsterdam, The Netherlands.

[†] These authors contributed equally to this manuscript.

* Corresponding author: t.noel@uva.nl

1. The most common hurdles to enter the field of Flow Chemistry

Despite the numerous advantages of flow chemistry, synthetic chemists may be hesitant to adopt this approach due to several reasons:

Problem 1 - Lack of familiarity: Many synthetic chemists are trained in traditional batch chemistry and may not be familiar with the principles and techniques of flow chemistry. This lack of familiarity can create a barrier to entry, making it difficult to persuade chemists to switch to flow chemistry.

Solutions:

- To facilitate the adoption of flow chemistry, it is essential to provide synthetic chemists with education and training on the most common principles and techniques of this approach. This could be achieved through workshops, seminars, and online courses. Additionally, incorporating flow chemistry into the undergraduate and graduate chemistry curricula could prove beneficial. A good understanding of the primary materials, setups, and individual components will help demystify the field of flow chemistry.
- Another important strategy is building solid and multidisciplinary scientific networks: collaborative efforts between flow chemistry experts and synthetic chemists can be highly valuable for sharing knowledge and expertise. Networking events, conferences, student exchanges, and online forums can all help foster such collaborations.
- Sharing success stories and case studies of flow chemistry's application to solve synthetic problems can help build familiarity and confidence in this approach. In fact, this is the core purpose of the present review.

Problem 2 - Limited infrastructure: Setting up a flow system requires specialized equipment and instrumentation, as well as modifications to the laboratory environment. This can pose a barrier for individual researchers, particularly those in academic settings, who may have limited resources or lack knowledge of what changes are needed to accommodate flow apparatuses. Additionally, synthetic chemists may be hesitant to acquire the necessary engineering skills required to utilize flow chemistry techniques. Technology is only beneficial when it provides support, not complications.

Solutions:

- Numerous companies have developed all-inclusive, multi-purpose flow reactors, which have made synthetic chemists' lives easier. These plug-and-play devices have reduced dimensions, enabling them to fit inside an ordinary fume hood, and allow chemists to focus primarily on the chemical transformation itself rather than on fittings, connections, and pumps.

- Collaborating with groups that already have flow chemistry equipment can significantly decrease the initial investment in infrastructure. This approach also offers opportunities for standardizing procedures, sharing expertise, and providing effective training for newcomers.

Problem 3 – Initial investment and costs: Arguably, the main obstacle to widespread adoption of flow chemistry within the synthetic chemistry community is the misconception that starting in this field is too costly. In fact, when reviewing the literature, one can quickly realize that every research group uses different materials for tubing, various arrangements of components, and custom-built reactors. In addition to funding concerns, another complication arises from not knowing what to purchase as an initial investment.

Solutions:

- Based on our years of experience in flow chemistry, we suggest that individuals interested in this fascinating field begin by collaborating with experts. This can provide a minimum set of skills and knowledge about materials and suppliers that are essential for success.
- 3D printing has become highly accessible and is commonly used to produce components required for flow chemistry using a 'Do-It-Yourself' approach. Although we recognize that 3D printing can have a positive impact on the costs associated with operating a flow lab, we also acknowledge that it may contradict the principles of standardization mentioned above. For instance, one lab may only print custom parts needed for a specific project, which may not be useful for someone new to the field. Nevertheless, by uploading designs and projects on public repositories following the principles of FAIR data, we could establish a thriving community where the most valuable and essential components are selected through a democratic process.

2. Getting Started with Flow Chemistry: Essential Accessories

In a final effort to decrease the activation energy for synthetic chemists willing to dive into the field of flow chemistry, we have created a list of essentials that an emerging investigator should consider purchasing. We also prepared some graphics representing reactors the investigator will be able to set up with the components listed: examples A and B are meant for a newbie, while example C is meant for a skilled investigator. Hopefully, this will help researchers to use a dedicated budget in the most effective manner possible.

Note: To ensure a fair cost evaluation, the lists below exclude purpose-built reactors such as photochemical or electrochemical reactors, which require a case-by-case assessment. In most situations, a suitable reactor can be obtained from existing market offerings, and the investigator can request a quote from the manufacturer. Alternatively, a DIY approach using 3D-printing can also be considered.

NEWBIE

Description:

The investigator has just been awarded a small grant (10 k€) to work on a research project based on flow chemistry. The investigator is still hesitant about the real benefits of flow chemistry in their research and just wants to provide the lab with a sufficient amount of fittings, tubing and equipment to complete the project. Once the project will be completed, the investigator will evaluate the possibility to purchase more materials and, in case, hire more people to keep working in the field of flow chemistry.

Expert advice:

For a newbie to the field of flow chemistry, the first purchase should be tubing and fittings - these are the building blocks of a flow reactor. But, we understand that choosing the right type and amount of tubing can be overwhelming. To make things easier, we recommend starting with 100 meters of PFA tubing (or FEP tubing if preferred) - it's flexible, heat-resistant, pressure-resistant and transparent, making it perfect for getting started. When it comes to fittings, the number of components needed will depend on the reactor network one wants to build. Ferrules, nuts, T-mixers, Luer adapters, and union bodies are just some of the fittings used to connect tubing segments, while BPRs and check valves are used to handle pressure (we suggest BPRs of 40 and 75 psi for a good compromise). Don't forget the tools - tube cutters and tweezers will come in handy. Overall, the cost of fittings is relatively low as tubing can be reused indefinitely as long as it remains in good condition, and fittings are usually made of PEEK, a chemically inert plastic material.

We recommend that newcomers to flow chemistry consider purchasing at least two syringe pumps, which are an essential part of the equipment but can also be quite expensive. However, as competition among brands has increased, prices have come down, making syringe pumps a more affordable option. They are also robust, require little maintenance, and can be used for a wide range of applications. One thing to keep in mind is that syringe pumps are limited in their ability to handle high pressure (typically less than 7 bar), but they are ideal for low flow rates (0.05-10 mL/min) and small volumes (1-20 mL), which makes them perfect for screening reaction conditions. Alternatively, one could opt for more expensive HPLC pumps, which can handle higher pressures (up to 80 bars), but they are typically better suited for larger volumes and may not be as accurate at low flow rates (< 0.1 mL/min).

SKILLED

Description:

The investigator has a previous experience in flow chemistry, e.g. as a visiting student in a flow chemistry group, and recognizes the benefits in their research. The investigator has just been awarded a grant of 30 k€ to work on new flow projects and would like to put the group (< 10 people) in the best position to run 2 or 3 projects in this field in parallel.

Expert advice:

For a skilled investigator looking for more flexibility and the ability to mentor several people in the field of flow chemistry, additional equipment and a larger budget are required. High-pressure and temperature sensors may be needed to perform reactions under these conditions with higher reproducibility, and we suggest purchasing stainless steel tubing with dedicated Swagelok connections to handle them. In addition to syringe pumps, we recommend investing in an HPLC pump and a peristaltic pump to handle large reaction scales, high pressures, slurries, and biphasic mixtures. A Mass-flow controller (MFC) is also essential for pumping gases in multiphase gas-liquid mixtures. Although MFCs are calibrated for specific gases, they can be used with other gases using mass-conversion factors. Finally, we suggest allocating a portion of the budget to purchase microreactors and microchips to enable fast mass and heat transfer in small channel reactors, which can be critical in certain situations (See review and, specifically, the sections on mass and heat transfer).

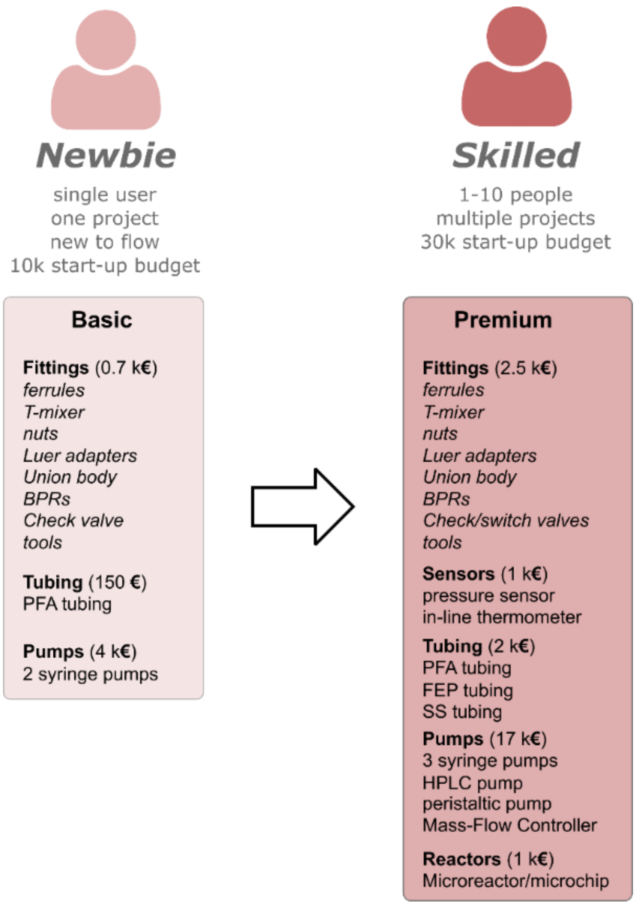

Picture	Name	Type
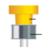	Super Flangeless™ Ferrule, Yellow ETFE/SS Ring, 1/16" OD Tubing, 1/4-28 or M6 Flat Bottom	P-259X
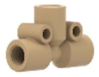	PEEK Low-Pressure Tee Assembly 1/16" PEEK .020 thru-hole (69 bar)	P-712
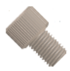	Super Flangeless™ Nut, Standard Knurl, Short, Natural PEEK, 1/32" or 1/16" OD Tubing, 1/4-28 Flat Bottom	LT-115X
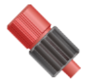	Luer Adapter Assembly 1/4-28 Female - Male, PEEK	P-655
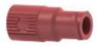	Luer Adapter 1/4-28 Female to Female Luer, PEEK	P-658
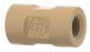	Union Body PEEK .020 thru hole, for 1/16" OD"	P-702-01
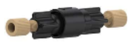	BPR Assembly 40 psi - Biocompatible (2.8 bar)	P-785
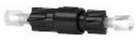	Check Valve Inline Assembly With Fittings	CV-3000
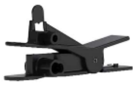	Polymer Tubing Cutter For 1/16" to 1/8" OD Tubing	A-327
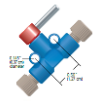	Shut-Off Valve Without Fittings, Blue ETFE, 10.0 µL	P-783
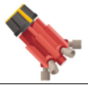	Injection Valve, 2 Postion-6-Port .063	V-540

Examples of setups

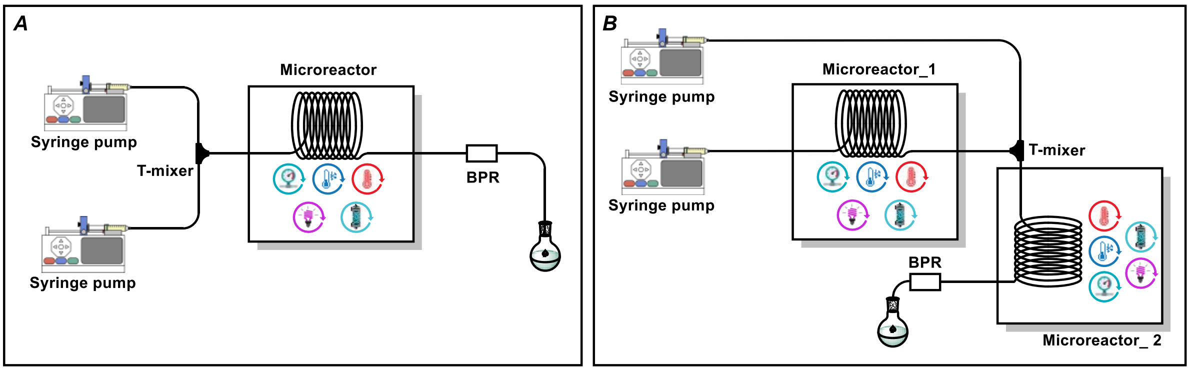

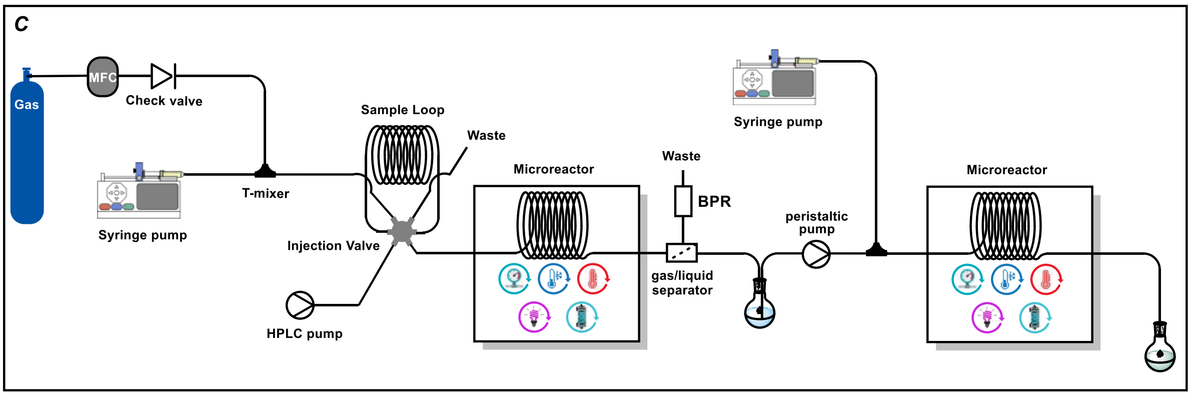

Supplement: SC-014-D3SC00992K-s001 [file SC-014-D3SC00992K-s001.pdf]
